# Nanocomposite Cryogels Based on Chitosan for Efficient Removal of a Triphenylmethane Dye from Aqueous Systems

**DOI:** 10.3390/gels11090729

**Published:** 2025-09-11

**Authors:** Maria Marinela Lazar, Claudiu-Augustin Ghiorghita, Daniela Rusu, Maria Valentina Dinu

**Affiliations:** “Petru Poni” Institute of Macromolecular Chemistry, Grigore Ghica Voda Alley 41A, 700487 Iasi, Romania; maria.lazar@icmpp.ro (M.M.L.); claudiu.ghiorghita@icmpp.ro (C.-A.G.); rusu.daniela@icmpp.ro (D.R.)

**Keywords:** composite cryogels, chitosan, natural zeolite, chrome azurol S, morphology, structural characterization, sorption isotherms

## Abstract

This work addresses the environmental challenge represented by persistent triphenylmethane dyes in aquatic systems through the development of chitosan (CS)–zeolite nanocomposite cryogels for the adsorption of chrome azurol S (CAS), as model dye. Nanocomposite cryogels were prepared via cryogelation at −20 °C with systematic variation in cross-linker concentration and zeolite content to modulate the network architecture and sorption performance. Comprehensive physicochemical characterization (SEM, EDX, FTIR) demonstrated that an intermediate cross-linker content (7.5 wt.% GA) combined with moderate zeolite loading (20 wt.%) yielded cryogels with the highest gel fraction yield and a homogeneous, highly interconnected macroporous structure. Swelling experiments at pH 1.2 revealed rapid water uptake equilibrium within 10 min, whereas adsorption isotherm analysis indicated that CAS sorption followed the Freundlich model, consistent with multilayer physical adsorption. The highest CAS adsorption capacity was achieved by CSGA5Z40 (250.81 mg g^−1^), indicating that low cross-linking combined with high zeolite loading maximizes uptake. These findings demonstrate that chitosan–zeolite nanocomposite cryogels are promising, reusable, and tunable adsorbents for sustainable removal of persistent dyes from wastewater.

## 1. Introduction

Over the past few decades, the release of synthetic dyes into aquatic environments has become an urgent environmental concern, with both ecological and public health ramifications. These dyes are extensively employed in industries such as textiles, printing, leather, paper, cosmetics, food processing, and agriculture due to their vibrant coloration, chemical stability, and resistance to degradation [1,2]. However, poor treatment practices and inefficiencies during industrial processes have resulted in significant contamination of freshwater systems.

The textile sector alone accounts for approximately 17–20% of global industrial water pollution [3], contributing substantially to dye-laden effluent discharge. Globally, over 700,000 tons of synthetic dyes are produced annually, and an estimated 1–15% of these dyes are released into the environment due to inefficient fixation during manufacturing and application processes [4]. Even at concentrations below 1 mg/L, dyes can severely impair water quality by inhibiting light penetration, thereby disrupting photosynthesis and aquatic biodiversity [5].

Synthetic dyes are typically categorized by either their application (e.g., acid, basic, reactive, mordant) or their chemical structure, which includes classes such as azo, anthraquinone, nitro, carbonyl, cyanine, and triphenylmethane [6]. Among these, triphenylmethane dyes—including crystal violet (CV), malachite green (MG), leucomalachite green (LMG), brilliant green, fuchsine, and chrome azurol S (CAS)—are widely used for their intense coloration, low cost, and broad applicability. However, their complex molecular structures and ionic characteristics make them highly resistant to conventional wastewater treatments.

The environmental persistence and toxicity of triphenylmethane dyes represent a significant ecological and health concern. Ecologically, their discharge into aquatic systems leads to intense coloration of water bodies, which reduces light penetration and consequently impairs photosynthetic activity in aquatic plants and microorganisms [7,8]. Biologically, these dyes are capable of crossing cellular membranes and accumulating in tissues, thereby increasing the risk of mutagenic, carcinogenic, and teratogenic effects [9]. Regulatory agencies have therefore established strict monitoring thresholds to control their presence in food and the environment. For example, the EU has set a Minimum Required Performance Limit of 2 µg/kg for the sum of MG and its metabolite leucomalachite green in aquaculture products. Similarly, the U.S. Food and Drug Administration monitors MG and CV, with detection limits as low as 1 µg/kg in edible tissues [10,11,12,13]. These stringent requirements highlight the necessity of developing effective and sustainable dye remediation technologies capable of meeting international safety standards.

A wide range of physicochemical and biological methods, including membrane filtration, electrocoagulation, flocculation, ion exchange, photocatalysis, and biological degradation, have been investigated for dye removal [14,15,16,17,18]. Despite their effectiveness, these techniques often suffer from high operational costs, complex infrastructure requirements, and the generation of secondary pollutants. Among these, adsorption has often used due to its low cost, simplicity, high efficiency, and lack of harmful byproduct formation [14,15,16,17,18]. While activated carbon remains the benchmark adsorbent due to its high surface area and strong affinity for dyes [19,20], issues related to high production cost, non-biodegradability, and poor regeneration limit its widespread application.

Consequently, attention has shifted toward the use of biodegradable and cost-effective adsorbents derived from natural polymers such as chitosan (CS), cellulose, alginate, xanthan gum, and pullulan [14,15,16,20]. These polysaccharides are abundant, renewable, and rich in functional groups like hydroxyl, carboxyl, and amine moieties, which enhance their binding affinity for dye molecules [14,15,16,20,21]. When structured into three-dimensional hydrophilic networks—commonly known as hydrogels—these materials demonstrate high water uptake capacity and potential for effective dye adsorption [4,14,15,16,22,23,24,25,26]. Nonetheless, conventional polysaccharide-based hydrogels often face limitations such as low surface area, weak mechanical properties, and reduced efficiency in real-world applications.

To overcome these constraints, composite hydrogels incorporating inorganic fillers have been developed. Materials such as zeolites [27,28], halloysite nanotubes [29], montmorillonite [30,31,32], laponite [33], magnetite [34,35,36], titanium dioxide [37], silica [38], and metal–organic frameworks [3,39] have been successfully embedded within the polysaccharide matrix to enhance porosity, mechanical stability, and adsorption performance. Zeolites, in particular, are attractive additives due to their high cation exchange capacity, negatively charged lattice structure, and ability to engage in both physical and chemical interactions with dye molecules.

The synergy between biopolymers and inorganic fillers enables the development of hydrogel nanocomposites with improved selectivity and adsorption capacity for specific dyes [27,28,29,30,31,32,33,34,35,36,37,38,39]. For example, incorporating zeolite into a carboxymethyl tamarind gum matrix has demonstrated significantly enhanced removal of crystal violet [28]. Despite such advances, there remain challenges in tuning hydrogel structures for optimal dye binding and ensuring durability over multiple adsorption–desorption cycles.

One emerging strategy to address these limitations is the application of cryogelation, a technique involving the freeze–thaw treatment of polysaccharide solutions to produce cryogels—macroporous, sponge-like materials with interconnected channels [40,41,42,43]. These structures formed with or without the addition of green cross-linkers, offer enhanced mechanical stability, fast mass transfer, and customizable physicochemical properties [40,41,42]. By adjusting cross-linking density, incorporating chelating functional groups, or embedding inorganic fillers, cryogels can be tailored for high-performance dye adsorption [17,40,41,43]. As a result, polysaccharide-based cryogels are emerging as a promising class of eco-friendly sorbents for the efficient removal of persistent dyes such as triphenylmethane compounds. Their tunable porosity, chemical versatility, and recyclability align well with the principles of green chemistry and circular water management.

Over the past several years, our research group has concentrated on the development of CS-based cryogel composites incorporating natural zeolite (clinoptilolite, CPL) for drug delivery applications [44], where varying the zeolite content enabled control over the internal structure, water uptake, and release profiles of bioactive compounds. Subsequently, we focused on environmental applications, applying ion-imprinting techniques to develop cryogels with specific recognition sites for selective heavy metal ion removal [45,46]. These materials showed enhanced sorption selectivity and capacity—particularly for Cu^2+^ and Co^2+^ ions—under both single- and multi-component systems. Their mechanical elasticity and chemical robustness enabled multiple reuse cycles without significant performance loss. We further expanded this concept by functionalizing the cryogels with aminopolycarboxylic acids, which significantly improved the sorption of Zn^2+^, Pb^2+^, Cd^2+^, Ni^2+^, and Co^2+^ in both batch [47] and fixed-bed column systems [48], demonstrating practical feasibility for continuous wastewater treatment. In another notable study [49], we applied Cu^2+^-ion imprinting and unidirectional ice-templating to create anisotropic cryogels capable of rapid and selective metal ion uptake from real photo-etching wastewater. These materials achieved over 90% removal efficiency for Cu^2+^, Ni^2+^, Fe^3+^, Zn^2+^, and Cr^3+^, confirming their high applicability under realistic operational conditions.

In the present study, we broaden the application scope of CS–zeolite nanocomposite cryogels from heavy metal ion removal to the adsorption of organic pollutants, specifically targeting triphenylmethane dyes. To the best of our knowledge, this is the first systematic investigation of the adsorption of chrome azurol S (CAS), a representative triphenylmethane dye, by CS–zeolite nanocomposite cryogels. We examine how variations in cross-linker concentration and zeolite content influence physicochemical properties, internal morphology, and CAS adsorption performance. This work thus expands the versatility of CS–zeolite nanocomposite cryogels as eco-friendly, high-efficiency adsorbents suitable for dye-contaminated wastewater.

## 2. Results and Discussion

CS-based nanocomposite cryogel beads were successfully fabricated via cryogelation at −20 °C, using glutaraldehyde (GA) as a cross-linker and natural zeolite as an inorganic filler. Briefly, an acidic CS solution was combined with a zeolite aqueous dispersion under continuous stirring to achieve uniform distribution of the filler. After equilibration, the mixture was cooled to 0 °C, followed by dropwise addition of GA solution under vigorous stirring to initiate cross-linking. The pre-gelled systems were then dispensed dropwise into liquid nitrogen, forming spherical cryogel beads. After complete freezing, the beads were transferred to a cryostat at −20 °C for 24 h to finalize the cryogelation process. Finally, beads were thawed, washed thoroughly with distilled water to remove residual reagents, and lyophilized.

**Figure 1:**
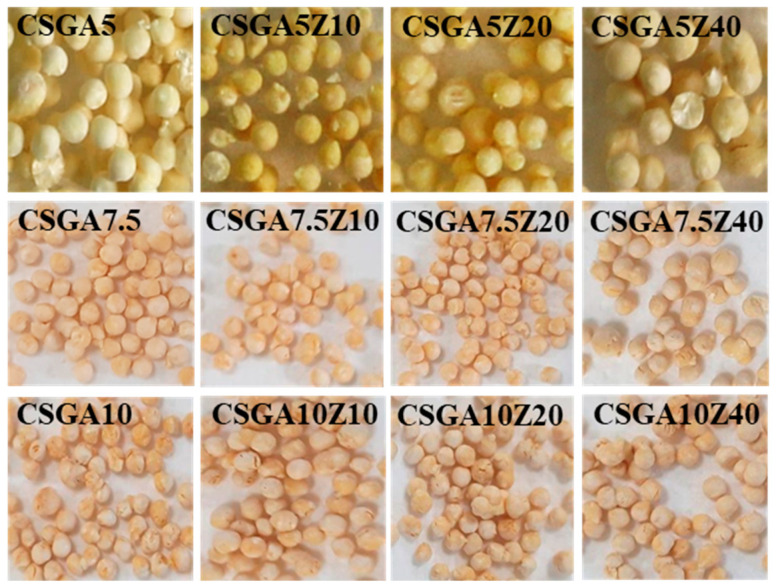
Optical images of the CSGA–zeolite nanocomposite cryogels with different cross-linking degrees and zeolite concentrations, illustrating the bead-shaped form and its variation with composition.

Optical images of the dried CSGA–zeolite nanocomposite cryogel beads (Figure 1) show that bead color changed from light yellow in the CSGA5 series to light brown in the more cross-linked samples (CSGA7.5 and CSGA10 series). This color shift reflects the formation of a higher number of imine bonds, corresponding to increased GA content. The bead dimensions varied between 2.57 and 3.03 mm (Table 1) depending on the composition of CSGA-based cryogels.

### 2.1. Effect of Cross-Linker Concentration and Zeolite Content on Network Formation

The gel fraction yield (GFY) data (Table 1) demonstrate a clear dependence of network formation efficiency on cross-linker concentration and zeolite content.

For CS cryogels without zeolite, increasing GA concentration from 5 wt.% to 10 wt.% decreased GFY from 86.00 ± 0.61% to 81.28 ± 0.97%. This suggests that high cross-linker content accelerates gelation kinetics, possibly leading to local phase separation and incomplete incorporation of polymer chains into the cryogel network.

In nanocomposite cryogels, zeolite addition modulated GFY differently, depending on GA concentration. At 5 wt.% GA, GFY remained relatively stable (~84–85%), with a slight decrease at 20 wt.% zeolite and partial recovery at 40 wt.%. This indicates that moderate zeolite amounts can enhance structural integrity, whereas high loading may cause particle agglomeration. At 7.5 wt.% GA, zeolite had a pronounced effect. GFY increased significantly, reaching a maximum of 88.17 ± 1.42% at 20 wt.% zeolite (CSGA7.5Z20). This suggests that zeolite acted as a nucleation center, facilitating more efficient cross-linking and uniform gelation. However, at higher zeolite content (40 wt.%), GFY dropped slightly, likely due to filler aggregation that disturbs the network formation. At 10 wt.% GA, GFY values stabilized (~81–82%) regardless of zeolite content, indicating that high cross-link density dominates network formation and diminishes the reinforcing effect of the filler.

These results reveal a synergistic effect between moderate GA concentration (7.5 wt.%) and intermediate zeolite loading (20 wt.%), yielding the highest GFY and suggesting optimal network uniformity.

### 2.2. Internal Morphology and Pore Size Districution of the Nanocomposite Cryogels

**Figure 2:**
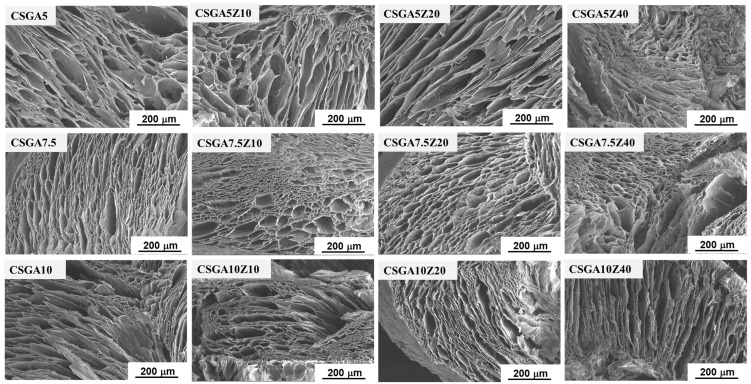
SEM micrographs of cross-sections of CSGA–zeolite nanocomposite cryogels prepared with different cross-linker ratios and zeolite contents, highlighting their internal morphology. Magnification: 150×; Scale bar: 200 µm.

**Figure 3:**
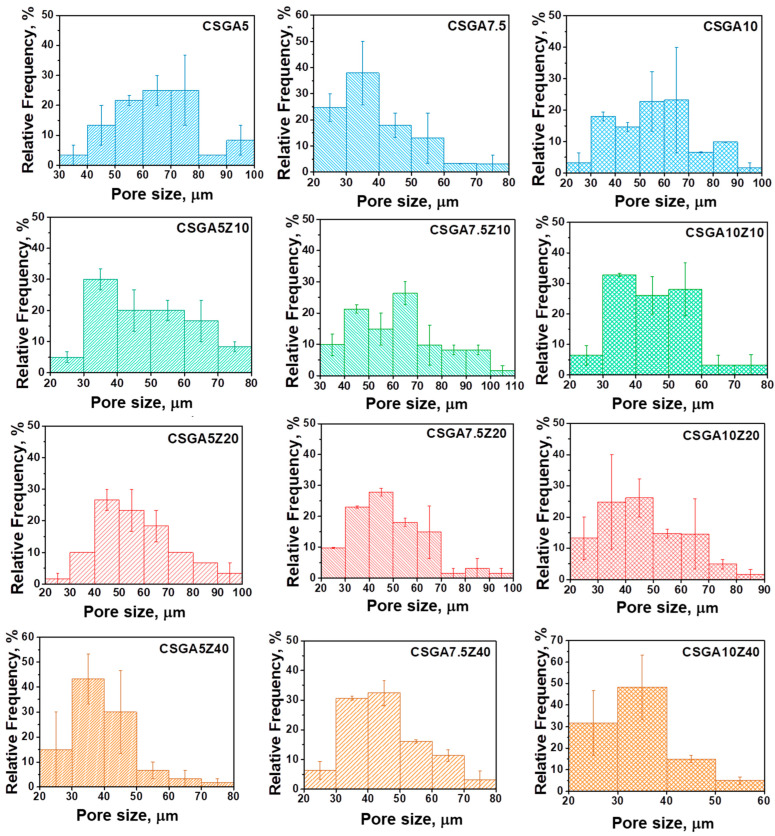
Histograms showing the pore size distribution of CSGA–zeolite nanocomposite cryogels with different cross-linker ratios and zeolite concentrations.

SEM analysis (Figure 2) and pore size distribution diagrams (Figure 3) provide complementary insights into the impact of composition on the cryogel microarchitecture.

At low GA content (5 wt.%), the pristine CSGA cryogels exhibit a characteristic lamellar morphology, consistent with structures formed through radial freezing of polysaccharide-based hydrogels [46]. Increasing the GA concentration to 7.5 wt.% leads to denser networks with thinner pore walls, reflecting enhanced cross-linking and more uniform gelation. At the highest cross-linking level of 10 wt.% GA, the cryogels develop compact, less porous, and mechanically rigid structures, indicative of rapid gelation kinetics. This result could also be correlated with the wide pore size distribution observed in the CSGA10 sample (Figure 3), which shows high polydispersity.

For the nanocomposite cryogels, zeolite particles are incorporated along the lamellar structures, but their influence varies with the GA concentration.

At 5 wt.% GA, zeolite additions up to 20 wt.% preserve the lamellar architecture, maintaining a relative frequency of ~28% of pores in the 40–50 μm range (Figure 3). However, at 40 wt.% zeolite, the cryogels show irregular, denser structures, with ~42% of pores sized 30–40 μm and ~30% between 40 and 50 μm, likely due to zeolite particle agglomeration. To confirm this result, SEM micrographs of CSGA5Z40 with a higher magnification are provided in the Supporting Information (Figure S1).

At 7.5 wt.% GA, moderate zeolite loadings (10–20 wt.%) increase the average pore size and improve pore uniformity (Figure 3). Notably, the CSGA7.5Z20 sample (7.5 wt.% GA, 20 wt.% zeolite) displays the most uniform and highly interconnected porous network (Figure 2), which correlates with the highest gel fraction yield (GFY) obtained in this series. The zeolite particles may stabilize the forming gel matrix during the freezing process, promoting improved pore connectivity and consistent pore wall formation. To further support this observation, SEM micrographs of CSGA7.5Z20 with magnifications of 500× and 1000×, respectively, are included in the Supporting Information (Figure S2).

At 10 wt.% GA, SEM micrographs show that the networks remain compact across all zeolite concentrations. At lower zeolite contents, the pore size distribution is highly polydisperse, but increasing zeolite content slightly reduces pore sizes. For instance, the CSGA10Z40 sample shows a relative frequency of ~50% of pores between 30 and 40 μm, and ~32% between 20 and 30 μm (Figure 3).

These findings clearly demonstrate that the interplay between cross-linker concentration and zeolite loading can be leveraged to tailor cryogel porosity and microstructure. Specifically, moderate cross-linking (7.5 wt.% GA) combined with intermediate zeolite content (20 wt.%) yields cryogels with the highest GFY, the most uniform and interconnected morphology.

**Figure 4:**
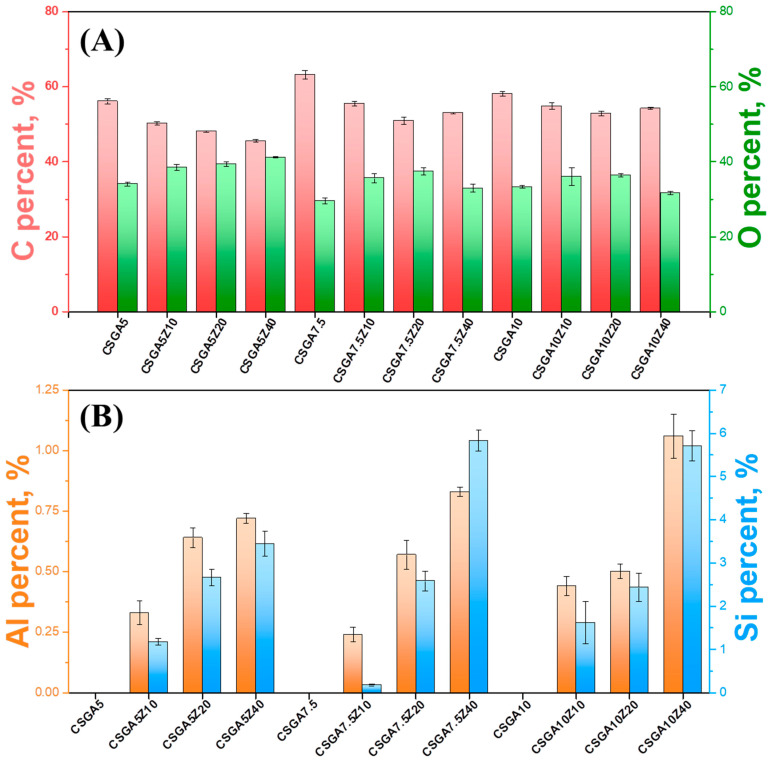
Elemental composition of CSGA–zeolite nanocomposite cryogels with varying cross-linker ratios and zeolite contents: (**A**) relative percentages of C and O, highlighting the decrease in C and corresponding increase in O upon zeolite incorporation; (**B**) relative percentages of Al and Si, showing progressive increases with higher zeolite loadings.

EDX analysis (Figures S3–S5, Supporting Information) revealed pronounced alterations in the elemental composition of CSGA-based cryogels upon zeolite incorporation. Pristine CSGA cryogels comprised predominantly C (~56–60%), O (~29–42%), and N (~6–9%), with variations linked to the cross-linking ratio. Increasing zeolite loading systematically reduced C content, attributed to substitution of C from the CSGA network by the zeolite framework, which introduced additional O along with Na, Mg, Al, Si, K, and Ca. For instance, O content increased from 34.09 ± 0.57% in CSGA5 to 41.20 ± 0.18% in CSGA5Z40 (Figure 4). Nitrogen levels remained stable across all nanocomposites, indicating preservation of N functionalities beneficial for surface activity and adsorption performance. The progressive increases in Si and Al content with higher zeolite loadings further confirm successful zeolite integration (Figure 4). For example, Si rises from 1.18 ± 0.07% in CSGA5Z10 to 3.44 ± 0.29% in CSGA5Z40, while Al increases from 0.33 ± 0.05% to 0.72 ± 0.02%.

### 2.3. Structural Characterization

**Figure 5:**
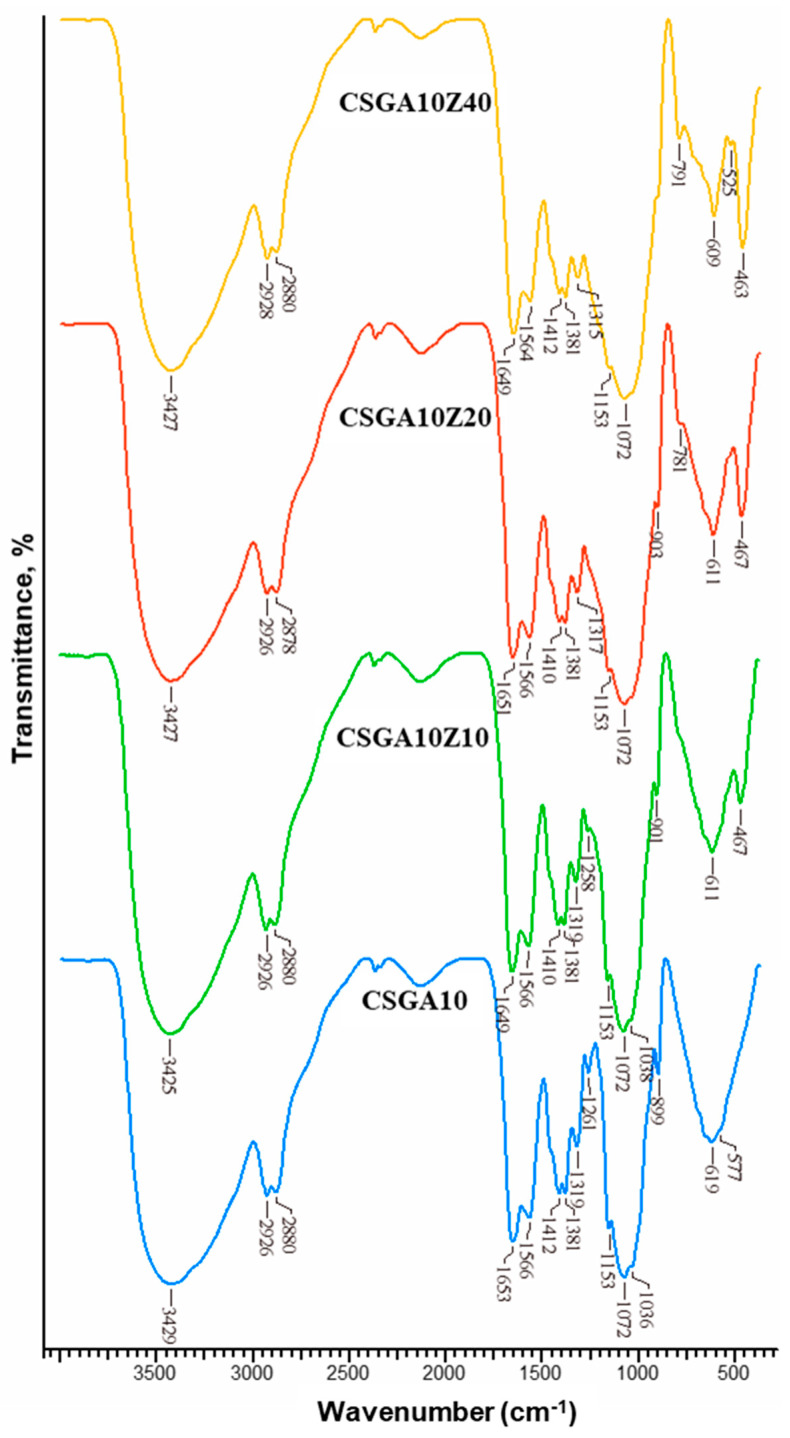
FTIR spectra of CSGA–nanocomposite cryogels cross-linked with 10 wt.% GA and containing different zeolite loadings: 10 wt.% (CSGA10Z10), 20 wt.% (CSGA10Z20), and 40 wt.% (CSGA10Z40).

FTIR spectroscopy was further employed to investigate the chemical structure of the CSGA cryogel beads cross-linked by GA and to evaluate the effect of zeolite incorporation (10–40 wt.%) on the network structure (Figure 5, Figures S6 and S7, Supporting Information). The FTIR spectra of CS and zeolite, as powders, are also provided for comparison in Figure S8 of the Supporting Information.

In the FTIR spectrum of CS (Figure S8, Supporting Information), several characteristic absorption bands were identified, consistent with the reported literature [46,50,51]. These include a broad band at 3464 cm^−1^ corresponding to O–H and N–H stretching vibrations, a band at 2874 cm^−1^ assigned to symmetric stretching of –CH_2_ groups, and a strong absorption at 1661 cm^−1^ attributed to the C=O stretching vibration of acetamide groups (amide I). Additional peaks were observed at 1599 cm^−1^ (NH_2_ bending), 1423 cm^−1^ (–CH_2_– bending), 1381 cm^−1^ (–CH bending), 1155 cm^−1^ (C–O–C asymmetric stretching), and at 1084 cm^−1^ and 1032 cm^−1^, associated with C3–OH and C6–OH stretching vibrations, respectively [50,51,52].

Upon cross-linking with GA, notable spectral changes were observed in the CS-based cryogels (Figure 5; Figures S6 and S7, Supporting Information). The most significant change was the disappearance of the –NH_2_ bending vibration band at 1599 cm^−1^, replaced by a new band at 1566 cm^−1^ in CSGA10 and at 1564 cm^−1^ in CSGA5 and CSGA7.5. According to previous studies [52,53], this modification indicates the formation of imine bonds (Schiff bases) between CS amino groups and GA aldehyde groups, confirming successful chemical cross-linking. Additionally, intensification of the bands around 1410–1412 cm^−1^, corresponding to –CH_2_ bending vibrations, is attributed to new methylene groups introduced by GA, further supporting cross-linking [52].

The incorporation of zeolite into the CSGA cryogel matrix led to further structural modifications detectable by FTIR (Figures S6 and S7, Supporting Information; Figure 5). Distinct absorption bands characteristic of zeolite (Figure S8, Supporting Information) were identified at 473 cm^−1^ and 608 cm^−1^ (Al–O stretching vibrations), and at 793 cm^−1^ and 1067 cm^−1^ (Si–O–Si stretching vibrations) [27,46]. These bands were present in all nanocomposite cryogels (Figure 5; Figures S6 and S7, Supporting Information), with their intensity increasing proportionally with zeolite content (especially in CSGA10Z40, CSGA7.5Z40, and CSGA5Z40 samples), confirming successful incorporation of the natural zeolite. Further spectral effects of zeolite addition included the broadening of the absorption region between 1072 and 1032 cm^−1^, where the strong Si–O stretching band from zeolite overlapped with C–O stretching vibrations of the CS anhydroglucose ring.

Importantly, no new peaks indicative of covalent bonding between CS chains and zeolite were detected, indicating that the zeolite particles are physically entrapped being in agreement with previous reports on the field [27,44].

### 2.4. Swelling Behavior

The swelling behavior of cryogels is a fundamental feature that underpins their successful use in diverse applications, including as adsorbents. Cryogels are known for the rapid diffusion of water within their network [54,55] due to their highly interconnected macroporous architecture, which minimizes resistance to fluid flow and facilitates efficient mass transport.

CS contains primary amino groups in its structure and behaves as a weak polyelectrolyte, meaning that the ionization of its amino groups depends on the pH of the medium [56]. At an acidic pH, its amino groups become protonated, thereby increasing the level of electrostatic repulsions between polymer chains. As the pH rises towards neutral or alkaline conditions, the amino groups gradually lose protons, leading to a decrease in charge density and a reduction in electrostatic repulsions between the CS chains.

**Figure 6:**
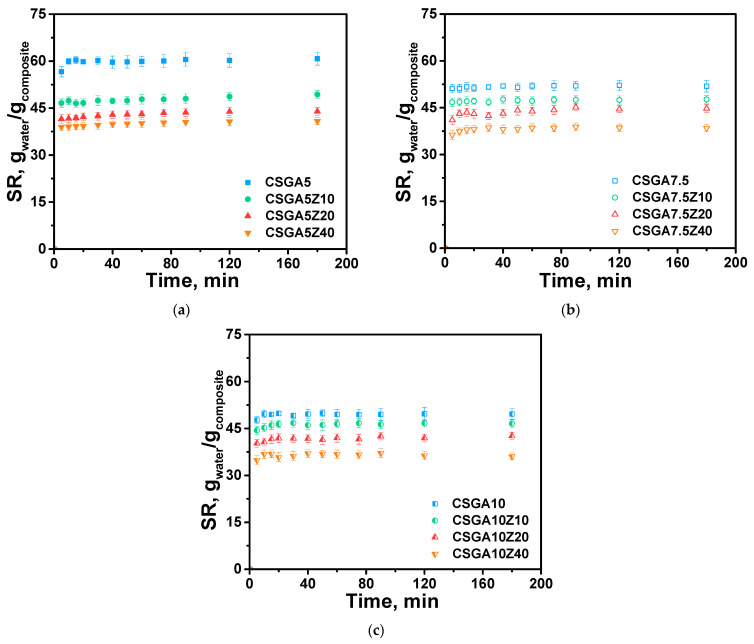
The swelling ratio of the CSGA–zeolite nanocomposite cryogels containing different zeolite loadings in a pH 1.2 aqueous solution: (**a**) CS-based cryogels with 5 wt.% cross-linking degree; (**b**) CS-based cryogels with 7.5 wt.% cross-linking degree; (**c**) CS-based cryogels with 10 wt.% cross-linking degree.

Based on these considerations, we investigated the swelling kinetics of the CSGA–zeolite nanocomposite cryogels in aqueous solution at pH 1.2 (Figure 6). Under these conditions, the amino groups of CS are fully protonated, thereby enabling maximum electrostatic repulsions between the polymer chains, which facilitate an increased water uptake.

As shown in Figure 6, all the nanocomposite cryogels reached their maximum swelling capacity within 10 min. This rapid swelling can be attributed to several factors, including the high external surface area of the cryogel beads, their highly porous inner architecture (Figure 2) with pores of several tens of micrometers enabling fast capillary diffusion of water inside the gel matrix (Figure 3), and the hydrophilic functional groups of chitosan (particularly the hydroxyl and protonated amino groups). The increase in cross-linker concentration and zeolite content within the CSGA cryogel beads enabled a decrease in the swelling capacity of the cryogels (Figure 6). This reduction can be attributed to the formation of more densely cross-linked polymer networks, resulting from the synergistic effects of chemical cross-linking between CS and GA, as well as the establishment of physical interactions (electrostatic attractions and hydrogen bonds) between CS and the zeolite particles. This combined mechanism restricts the mobility of polymer chains and reduces the expansion of the cryogel matrix upon hydration.

To gain insight into the water uptake mechanism, the experimental swelling data have been fitted with the nonlinear forms of the Pseudo-First Order (PFO) (Equation (1)) and Pseudo-Second Order (PSO) (Equation (2)) kinetic models [57,58].

(1) $$q_{t}=q_{e}(1-e^{-k_{1}t})$$

(2) $$q_{t}=\frac{k_{2}q_{e}^{2}t}{1+k_{2}q_{e}t}$$

where *q_e_* and *q_t_* (g/g) denote the water adsorption capacities at equilibrium and at a specified time, respectively, *k*_1_ (min^−1^) represents the PFO kinetic model rate constant, and *k*_2_ (g/g·min) corresponds to the rate constant of the PSO kinetic model. The parameters derived from fitting the water absorption data to these kinetic models are presented in Table 2.

The PSO kinetic model provided slightly higher coefficients of determination (R^2^ = 0.997–0.999) compared to PFO model, indicating that it more accurately describes the water absorption kinetics of the CSGA–zeolite nanocomposite cryogels. The superior fit to the PSO model suggests that the rate-limiting step of the absorption process is likely governed by chemisorption mechanisms, involving valence forces through the exchange of electrons between the functional groups of CS and water molecules. This finding is consistent with previous studies reporting that water uptake in superabsorbent composites is not solely controlled by physical diffusion, but rather by chemisorption. For example, Xu et al. [57] demonstrated that the swelling of poly(acrylic acid)-grafted composites prepared from dopamine-coated waste sea buckthorn branches followed PSO kinetics, while Jayanudin et al. [58] similarly observed PSO-controlled swelling in CS–graft–poly(acrylic acid) hydrogels. In line with these reports, the present results confirm that the swelling and absorption behavior of CSGA–zeolite nanocomposites is predominantly driven by chemisorption rather than simple Fickian diffusion.

### 2.5. Adsorption of CAS Dye

#### 2.5.1. Sorption Isotherms

**Figure 7:**
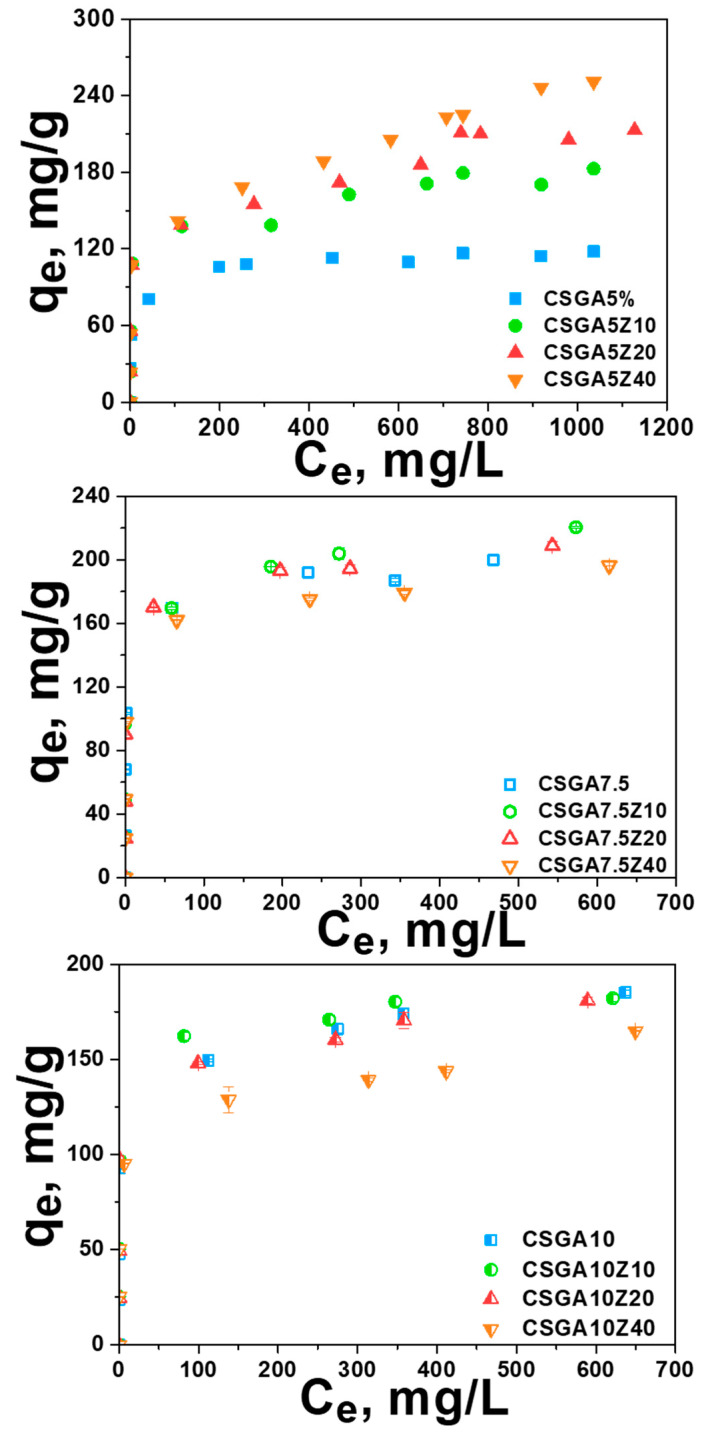
Effect of equilibrium concentration on CAS adsorption by the CSGA–zeolite nanocomposite cryogels with different cross-linker ratios and zeolite content. Sorption conditions: T = 298 K; pH = 5.3; V = 0.01 L; sorbent dose = 2 g L^−1^; initial CAS concentration ranging from 50 to 1200 mg L^−1^.

The sorption capacity of CS–zeolite nanocomposite cryogels was evaluated under batch equilibrium conditions. Figure 7 illustrates the effect of equilibrium concentration on CAS adsorption by the CSGA–zeolite nanocomposite cryogels.

In order to elucidate which isotherm describes our equilibrium data better, the nonlinear forms of Langmuir, Freundlich, and Dubinin–Radushkevich (D-R) isotherms were applied (Figures S9–S11, Supporting Information). The nonlinear regression method was adopted because it was demonstrated to be a better approach to evaluate the isotherm parameters [59]. The nonlinear form of the Langmuir isotherm [60] is described by Equation (3):

(3) $$q_{e}=\frac{q_{m}K_{L}C_{e}}{1+K_{L}C_{e}}$$

where *q_e_* is the amount of the dye sorbed per gram of sorbent at equilibrium (mg/g), *q_m_* is the maximum sorption capacity of dyes (mg/g), *C_e_* is the concentration of dye solution at equilibrium (mg/L), and *K_L_* is the Langmuir constant (L/mg).

The Freundlich model explains heterogeneous adsorption where heat distribution is non-uniform and is expressed by Equation (4) [61]:

(4) $$q_{e}=K_{F}C_{e}^{1/n}$$

where *K_F_* is the Freundlich constant (mg^1−1/*n*^·L^1/*n*^·g^−1^), and 1/*n* is a parameter related to the heterogeneous distribution of active sites on the sorbent surface. If 1/*n* < 1, bond energies increase with the surface density; if 1/*n* > 1, bond energies decrease with the surface density; and if 1/*n* = 1, all surface sites are equivalent.

The D-R isotherm is generally illustrated by Equation (5) [59]:

(5) $$q_{e}=\;q_{DR}exp\left\{-\beta {\left[RT\ln\left(1+\frac{1}{C_{e}}\right)\right]}^{2}\right\}$$

where *q_DR_* is the maximum dye sorption capacity(mg/g), *K_DR_* is the D-R isotherm constant (mol^2^/kJ^2^), *β* is the D-R isotherm constant (mol kJ^−1^)^2^, *T* is the absolute temperature (K), and R is the gas constant.

The D-R isotherm constant, *β*, is related to the mean free energy of sorption, *E* (kJ mol^−1^), and the value of *E*, calculated by Equation (6), is used to estimate the type of sorption [59]:

(6) $$E=\frac{1}{{\left(2K_{DR}\right)}^{1/2}}$$

when the value of *E* is between 8 and 16 kJ mol^−1^, the sorption mechanism is described by an ion exchange process, while the values of *E* < 8 kJ mol^−1^ indicate a physical sorption.

The maximum amount of dye that is retained onto sorbents is evaluated from sorption isotherms. Langmuir, Freundlich, and D-R isotherm profiles for the adsorption of CAS by CSGA nanocomposite cryogels with different zeolite contents are shown in Figures S9–S11 (Supporting Information), and the values of the isotherm parameters obtained from nonlinear regression methods are listed in Table 3, Table 4 and Table 5, respectively.

The values of the regression coefficient of determination (R^2^), located in the range 0.732–0.964, and the low values of the nonlinear chi-square test (χ^2^) show that the Freundlich isotherm is suitable for the sorption process of CAS dye onto the CSGA–zeolite nanocomposite cryogels investigated in this work. The lower values of R^2^ and the high values of χ^2^ found by fitting the D−R isotherm on the experimental data indicate that this isotherm does not describe the sorption of the CAS dye onto the CSGA–zeolite nanocomposite cryogels well. The low values of *E* (<8 kJ moL^−1^), estimated taking into consideration the D-R isotherm constant, *β*, reveal the physical sorption as more probable mechanism for the sorption process onto CSGA–zeolite nanocomposite cryogels. This mechanism indicates that the sorption process is illustrated by the formation of weak physical attraction forces, such as hydrogen bonding, electrostatic interactions, and van der Waals forces, between dye molecules and active sites of CSGA–zeolite nanocomposite cryogels. The results obtained indicate that the sorption of CAS dye onto the surface and internal pores of all cryogels is multilayer adsorption, which demonstrates that this process follows the Freundlich isotherm [62].

#### 2.5.2. Mechanism of CAS Sorption

**Figure 8:**
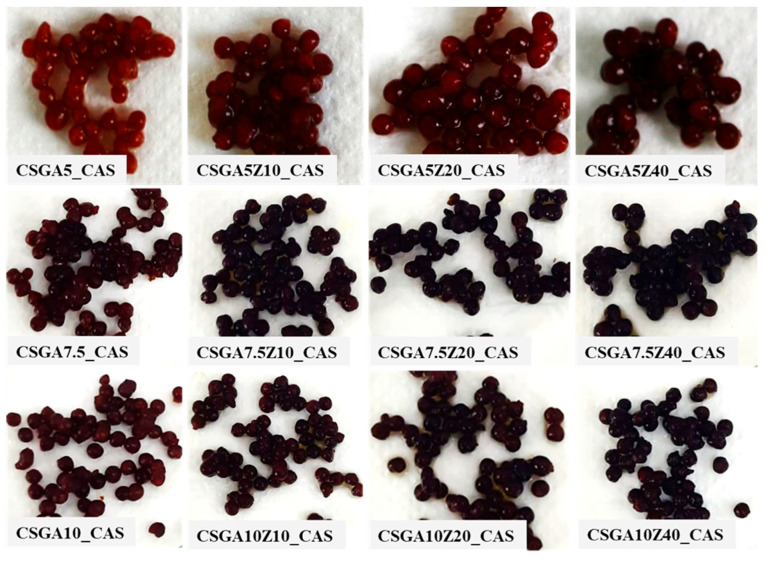
Optical images of CSGA nanocomposite cryogels with varying cross-linker ratios and zeolite contents in their swollen state after CAS dye sorption.

Figure 8 shows the CSGA nanocomposite cryogels with different cross-linker ratios and zeolite contents in swollen states, after the sorption of the CAS dye.

As can be observed in Figure 8, CAS dye has been uniformly distributed in the interior of the CSGA nanocomposite cryogels, and this also supports the uniform distribution of the active sites in the nanocomposite cryogels.

**Figure 9:**
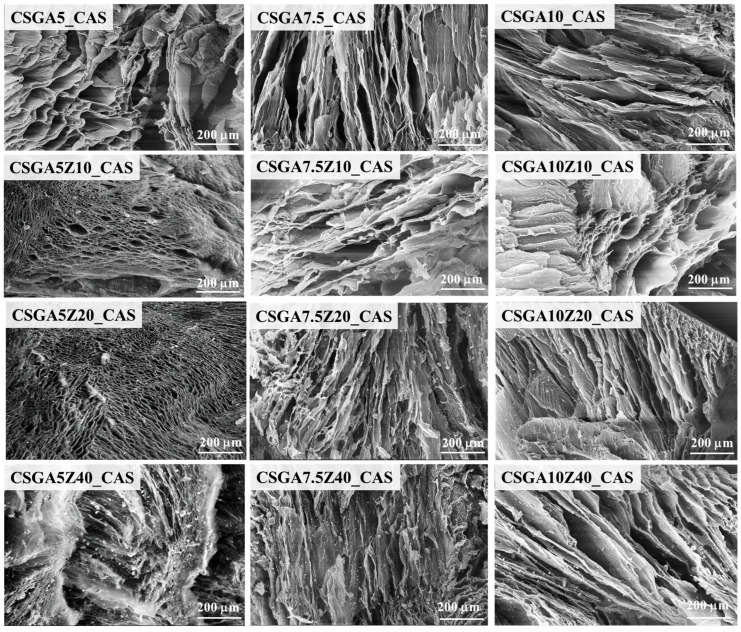
SEM micrographs of cross-sections of CAS-loaded CSGA–zeolite nanocomposite cryogels with different cross-linker ratios and zeolite content. Scale bar: 200 µm.

According to the discussion of isotherm modeling results, it can be proposed that the adsorption process of CAS involves electrostatic and hydrogen bonding with the functional group of the CS network as well as ion exchange with the zeolite surface. In order to understand the interaction mechanism of the CSGA-based cryogels with CAS dye, SEM (Figure 9) and FTIR analyses were performed after CAS sorption on CSGA-based cryogels. The SEM micrographs reveal distinct structural changes depending on the GA content and zeolite incorporation (Figure 9), highlighting how both parameters modulate the dye adsorption behavior and structural stability of the cryogels.

For the 5 wt.% GA cross-linking series (CSGA5), SEM images show that the cryogel without zeolite retains a relatively open, lamellar pore structure. However, in the case of CSGA5Z10 and CSGA5Z20, there is a drastic decrease in pore size, with pore walls appearing thicker and denser (Figure 9, first column). Notably, CSGA5Z40—containing the highest zeolite content—exhibits almost complete pore collapse, with the disappearance of well-defined porous features. This dramatic densification strongly suggests intense interactions between the dye molecules and both the CS matrix and the zeolite phase, leading to structural rearrangement or even partial clogging of the pores, supporting a high dye uptake capacity, as shown above (250.81 mg g^−1^ for CSGA5Z40, Table 3).

For the 7.5 wt.% GA cross-linking series (CSGA7.5), the SEM images demonstrate that the lamellar morphology is largely preserved after CAS sorption (Figure 9, middle column). This stability is more evident in the CSGA7.5Z20 sample, which prior to sorption exhibited the most uniform and interconnected pore network (Figure 2) and the highest GFY (Table 1). The ability of the CSGA7.5Z20 sample to retain its ordered lamellar structure after dye uptake highlights the synergistic role of moderate cross-linking and optimal zeolite dispersion in providing both sorption capacity and structural robustness.

The 10 wt.% GA cross-linking series (CSGA10) also displays remarkable morphological stability after dye interaction. SEM images show that the lamellar architecture of these highly cross-linked cryogels is largely preserved across all zeolite loadings (CSGA10Z10, CSGA10Z20, and CSGA10Z40), with well-defined, compact lamellar walls still clearly visible (Figure 9, last column). The thicker, highly cross-linked polymer network in these samples imparts mechanical rigidity, preventing collapse or pore distortion even after CAS sorption. SEM observations confirm that the cross-linking degree is the dominant factor governing cryogel structural resilience during dye uptake. Despite the highly polydisperse pore size distributions observed in CSGA10 cryogels, the preserved lamellar architecture and robust network ensure efficient mass transport, indicating that pore size variability does not create diffusion barriers. Thus, cross-linking degree emerges as the key determinant of both structural stability and sorption properties. Comparable findings have previously been reported for the sorption of CV and Methylene Blue (MB) onto dextran-based cryogels [43], for the sorption of MB and Congo Red (CR) onto CS/carboxymethyl cellulose hydrogels [63], and for the sorption of CR onto gelatin-based hydrogels [64].

EDX analysis of the CSGA-based cryogels (Figures S12–S14, Supporting Information) revealed that the atomic percentages of Si and Al remained unchanged under sorption conditions, confirming the structural stability of the natural zeolite within the CSGA matrix. Furthermore, the percentages of Ca^2+^ and K^+^ were almost constant following CAS sorption (Table S1, Supporting Information). In addition, the EDX spectra obtained after CAS dye adsorption revealed the appearance of sulfur (S), originating from the dye, as shown in Figures S12–S14 (Supporting Information). This result confirms the successful incorporation of CAS dye into the CSGA-based cryogel matrix.

**Figure 10:**
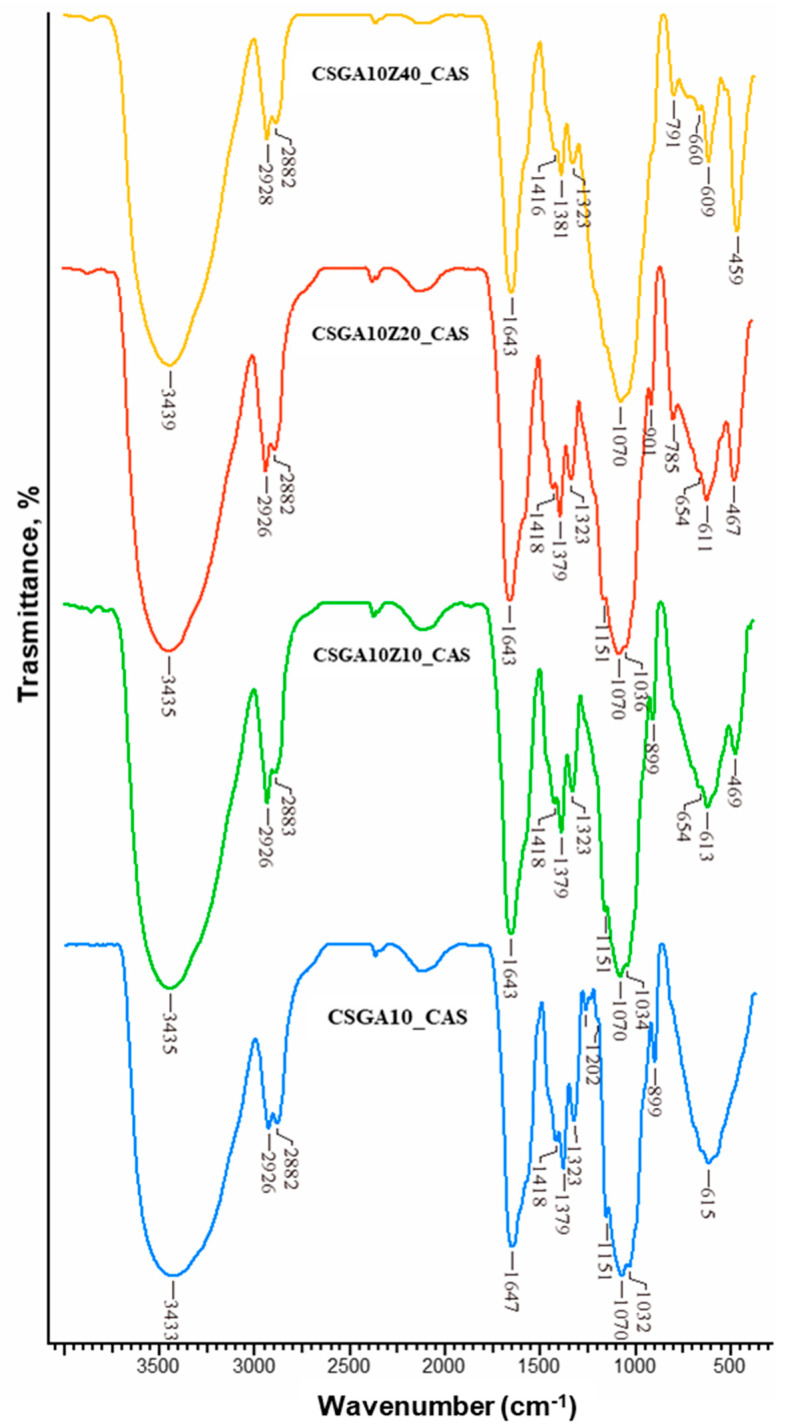
FTIR spectra of CAS-loaded CSGA10 nanocomposite cryogels with varying zeolite contents (10, 20, and 40 wt.%). The characteristic absorption bands corresponding to the functional groups of the CSGA network, zeolite framework, and CAS dye are labeled.

FTIR studies (Figure 10 and Figures S15 and S16, Supporting Information) were also performed to further understand the sorption mechanism of CAS dye by CSGA-based cryogels. Compared to the FTIR spectrum of the pristine CSGA10 cryogel (Figure 5) after the sorption of CAS (sample CSGA10_CAS) (Figure 10), the following spectral changes were observed:

(i) A red-shift of the –OH/–NH stretching from 3429 to 3433 cm^−1^ indicates formation of hydrogen bonds between CAS dye molecules and –OH/–NH groups of the cryogel matrix.
(ii) A blue-shift of the amide I band from 1653 to 1647 cm^−1^ suggests direct involvement of C=O groups in dye binding, possibly through dipole–dipole or hydrogen bonding interactions.
(iii) A down-shift of the band at 1566 cm^−1^ is attributed to the imine bond stretching vibrations (just a shoulder in the spectra of CSGA10_CAS).
(iv) A down-shift and a red-shift of the –CH_2_ bending band from 1412 to 1418 cm^−1^ are observed.
(v) The intensity of the –CH bending band at 1379 cm^−1^ increases.
(vi) A blue-shift of the C6–OH stretching band from 1036 to 1032 cm^−1^ points to participation of hydroxyl groups in binding with the dye.

For the CSG–zeolite nanocomposite cryogels, similar trends were observed. After CAS sorption (CSGA10Z20_CAS), the spectrum showed the following:

(i) A red-shift of the –OH/–NH band from 3427 to 3435 cm^−1^, suggesting even stronger hydrogen bonding interactions facilitated by the zeolite-modified surface;
(ii) A blue-shift of the amide I band from 1651 to 1643 cm^−1^, further supporting involvement of C=O in dye binding;
(iii) A down-shift of the band at 1566 cm^−1^, attributed to the imine bond stretching vibrations (just a shoulder in the spectra of CSGA10Z20_CAS);
(iv) A down-shift and red-shift of the –CH_2_ bending from 1410 to 1418 cm^−1^;
(v) Increased intensity of the –CH bending at 1379 cm^−1^;
(vi) A decrease in the intensity of the Al–O bands at 467 and 611 cm^−1^, observed after dye sorption, indicating that the zeolite’s surface –OH and Al–O groups are directly involved, likely via an ion-exchange mechanism.

Similar spectral shifts were also observed in the CSGA5 and CSGA7.5 series, both with and without zeolite (Figures S15 and S16, Supporting Information).

Overall, these comparative FTIR results before and after dye sorption clearly demonstrate that the CAS dye interacts strongly with: –OH and –NH groups, through hydrogen bonding; C=O and C=N groups, via dipole–dipole interactions, and with zeolite surface –OH and Al–O sites, through an ion-exchange mechanism.

#### 2.5.3. Effect of Competing Ions on CAS Sorption

**Figure 11:**
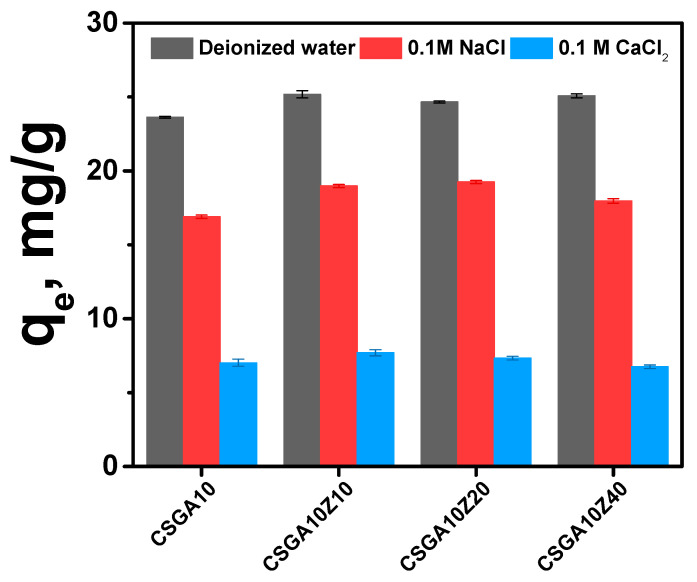
Effect of competing ions on CAS sorption by the CSGA10 nanocomposite cryogels with different zeolite contents. Sorption conditions: T = 298 K; pH = 5.3; V = 0.01 L; sorbent dose = 2 g L^−1^; initial CAS concentration = 50 mg L^−1^.

The effect of common competing ions on the sorption performance of CSGA10-based nanocomposite cryogels with varying zeolite contents is presented in Figure 11.

In deionized water, all samples exhibited the highest sorption capacity (~24–26 mg g^−1^), indicating that in the absence of ionic competition, the functional groups of the CSGA network and the surface of the zeolite particles are fully available for CAS uptake.

In the presence of monovalent Na^+^ ions (0.1 M NaCl), the sorption capacity decreased to ~18–19 mg g^−1^ across all samples. This reduction can be attributed to the electrostatic shielding of negatively charged CAS molecules, as Na^+^ ions compete with cationic binding sites on the cryogel matrix, thereby reducing the effective electrostatic interactions that result in CAS adsorption.

A more pronounced inhibitory effect was observed in the presence of bivalent Ca^2+^ ions (0.1 M CaCl_2_), where the sorption capacity decreased to ~6–7 mg g^−1^. The strong suppression of CAS absorption is probably due to two synergistic effects: (i) Ca^2+^ ions exhibit stronger electrostatic interactions with the negatively charged fragments of CAS, outperforming dye molecules in terms of adsorption sites, and (ii) Ca^2+^ ions may promote ion exchange with the zeolite surface, thereby reducing the accessibility of active sorption sites. Our results are consistent with previous reports on dye adsorption onto montmorillonite [65] and ion-exchange resins [66], which also showed reduced sorption capacity in the presence of competing ions such as Na^+^ and Ca^2+^.

Overall, these findings demonstrate that although CSGA10-based nanocomposite cryogels exhibit high sorption performance in deionized systems, their efficiency can be significantly reduced in saline environments. The strong inhibitory effect of Ca^2+^ highlights the importance of considering water composition in practical dye removal applications, particularly in natural waters or wastewater streams rich in divalent cations.

#### 2.5.4. Desorption and Reusability Studies

The desorption and reusability of sorbent materials is a critical factor in evaluating their practical applicability for wastewater treatment [27,67].

The desorption of CAS dye from the CSGA–zeolite nanocomposite cryogels was successfully achieved through a two-step chemical regeneration protocol combining acidic/organic and alkaline treatments. The initial HCl–methanol step effectively weakened electrostatic and hydrophobic interactions, while the subsequent NaOH treatment promoted deprotonation of functional groups, facilitating the release of strongly bound CAS molecules. This sequential process ensured efficient dye removal and restored the cryogels sorption capacity, enabling their reuse in multiple adsorption–desorption cycles.

**Figure 12:**
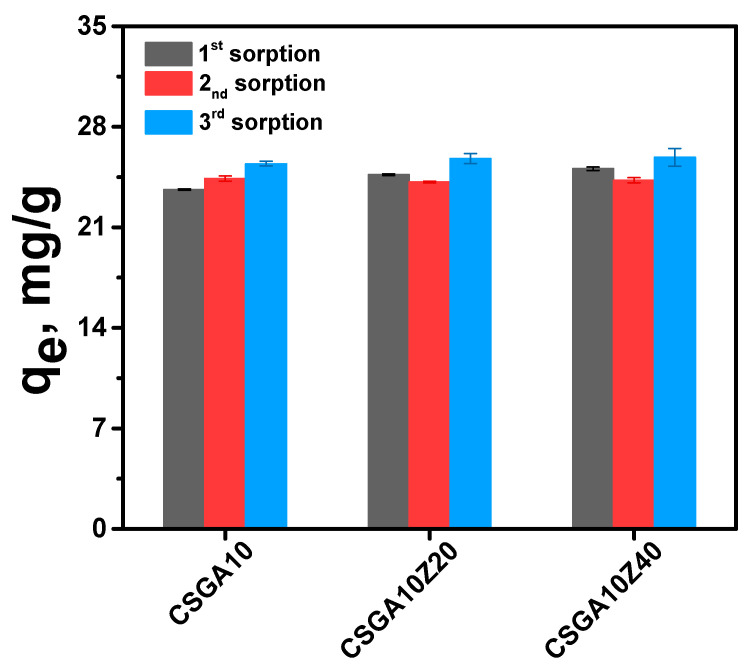
Reusability performance of CSGA10 nanocomposite cryogels with different zeolite contents in CAS sorption. Sorption conditions: T = 298 K; pH = 5.3; V = 0.01 L; sorbent dose = 2 g L^−1^; initial CAS concentration = 50 mg L^−1^.

Figure 12 shows the reusability performance of CSGA10-based nanocomposite cryogels without (CSGA10) and with different zeolite contents (20 and 40 wt.%) over three consecutive sorption–desorption cycles.

In the first cycle, all cryogels exhibited high sorption capacities (~24–26 mg g^−1^), consistent with the batch equilibrium studies (Figure 11). After regeneration, the cryogels maintained a significant fraction of their initial adsorption performance, with only a moderate decline in capacity observed across subsequent cycles. Importantly, the incorporation of zeolite did not lead to a significant loss in chemical stability, as all nanocomposites retained their structural integrity after repeated regeneration. Moreover, the FTIR spectra recorded for the CSGA-based sorbents (Figure S17, Supporting Information) demonstrated that the structural integrity of the sorbents remained unchanged under the experimental conditions applied for CAS desorption and sorbent regeneration. In the spectra of the CSGA10-based nanocomposites, the characteristic absorption bands corresponding to CS cross-linked with GA, as well as those associated with the zeolite, were clearly observed at similar wavenumber positions (Figure S17, Supporting Information).

These findings highlight the robustness and reusability of CSGA10-based nanocomposite cryogels for dye removal applications.

## 3. Conclusions

This study reports the preparation CS–zeolite nanocomposite cryogel as beads for CAS adsorption. Gel fraction yield and SEM analyses revealed a strong interplay between cross-link density and filler content. Moderate GA concentration (7.5 wt.%) combined with intermediate zeolite loading (20 wt.%) produced the highest GFY (88.17 ± 1.42%), the most uniform pore size distribution, and an interconnected lamellar network. In contrast, excessive cross-linking (10 wt.% GA) yielded compact, less porous structures, while high zeolite content (40 wt.%) caused filler agglomeration and network irregularities.

FTIR and EDX confirmed successful covalent cross-linking of CS chains via Schiff base formation and physical incorporation of natural zeolite particles. Increasing zeolite content led to proportional rises in Si and Al signals without evidence of new covalent bonds, indicating that the filler is physically entrapped within the polymer matrix.

All cryogels exhibited rapid water uptake at pH 1.2, reaching equilibrium within 10 min due to their interconnected macroporosity and hydrophilic functional groups. Increasing GA and zeolite content reduced swelling ratios through combined chemical and physical cross-linking effects that restricted chain mobility.

CAS adsorption capacities varied from 117.95 mg g^−1^ for CSGA5 to 250.81 mg g^−1^ for CSGA5Z40, indicating that high zeolite content at low GA cross-linking maximizes sorption capacity. In contrast, higher cross-linker content generally reduced dye uptake despite structural stability improvements. The Freundlich model was the isotherm that best described the equilibrium data, confirming multilayer adsorption on a heterogeneous surface. Dubinin–Radushkevich analysis yielded *E* < 8 kJ mol^−1^, supporting a physical adsorption mechanism dominated by hydrogen bonding, electrostatic interactions, and van der Waals forces. The SEM and FTIR analyses after dye sorption showed that CAS molecules interact with –OH, –NH, C=O, and C=N groups of the CS network, as well as with zeolite –OH and Al–O sites via ion exchange.

The CSGA–nanocomposite cryogels’ sorption efficiency is significantly reduced in saline environments, especially in the presence of Ca^2+^. However, they can be effectively regenerated by a two-step protocol, retaining most of their sorption capacity and structural integrity over three reuse cycles.

In summary, optimal performance requires balancing cross-link density and zeolite dispersion to achieve high porosity, structural stability, and abundant accessible adsorption sites. These findings establish design guidelines for tailoring bio-based nanocomposite cryogels as environmentally sustainable adsorbents for the remediation of dye-polluted wastewater.

## 4. Materials and Methods

### 4.1. Materials

Chitosan (CS), as a powder with a molar mass of 341 kDa and a deacetylation degree (DD) of 85%, was purchased from Sigma-Aldrich (Darmstadt, Germany) and used without further purification. The CS molar mass was calculated from the intrinsic viscosity of CS dissolved in 0.3 M CH_3_COOH–0.2 M CH_3_COONa (1:1, *v*/*v*), at 25 ± 0.1 °C [68]. Glutaraldehyde (GA), as an aqueous solution with a concentration of 25% (*w*/*w*) in distilled water, was also purchased from Sigma-Aldrich and was used as a cross-linking agent. Natural zeolite, containing 60–70% CPL, was obtained from the volcanic tuffs cropped out in the Macicas area (Cluj County, Romania) and was used as inorganic filler within the CS network. CAS dyes from Sigma-Aldrich were used after being recrystallized three times from an aqueous methanol solution (methanol/water, 70/30, *v*/*v*). Glacial acetic acid (≥99.8%), methanol (≥99.9%), and hydrochloric acid (37%, *v*/*v*), purchased from Chemical Company (Iasi, Romania), were used as received. Sodium hydroxide (≥98.0%), sodium chloride (≥99.0%) and calcium chloride (≥93.0%) were purchased from Fluka Chemie GmbH (Buchs, Switzerland) and used as received.

### 4.2. Methods

#### 4.2.1. CS-Based Nanocomposite Cryogels

CS-based composite cryogel beads were prepared by cryogelation at −20 °C, using GA as a cross-linker and natural zeolite as an inorganic filler. Briefly, 10 g of a 3 wt.% CS solution in 2% (*v*/*v*) acetic acid was mixed for 1 h with 4.58 mL of distilled water containing dispersed zeolite (37.5, 75, or 150 mg). After homogenization, the mixtures were cooled to 0 °C in an ice-water bath, and 0.42 mL of GA solution (5, 7.5, or 10 wt.%) was added dropwise under vigorous stirring for 1 h. The pre-gelled mixtures were then poured dropwise into liquid nitrogen, forming spherical beads. Following complete freezing, the beads were transferred to a cryostat at −20 °C and kept for 24 h to complete the cryogelation process. The resulting cryogel beads were thawed at room temperature for 1 h, extensively washed with distilled water (~900 mL) to remove unreacted reagents, and finally dried by lyophilization (Freeze Dryer Biobase BKFD10S; Jinan, China) at −60 °C and 10 Pa for 48 h. The blank samples (CSGA5, CSGA7.5, and CSGA10) were prepared and dried in the same manner, but without the addition of zeolite.

#### 4.2.2. Gel Fraction Yield

The transformation of reactants in CSGA–zeolite beads was evaluated by determining the gel fraction yield (GFY, %), using Equation (7):

(7) $$\mathrm{GFY},\;\%\;=\frac{W_{d}}{\;W_{m}}\;\times 100\;$$

where *W_d_* (g) is the weight of dried CS-based nanocomposite cryogels (under a vacuum in the presence of P_2_O_5_ until constant weight), and *W_m_* (g) represents the weight of all used reactants. The gel content test was repeated three times for each cryogel, and the average value was reported.

#### 4.2.3. FTIR Spectroscopy

Fourier transform infrared (FT-IR) spectroscopy was first used to identify the chemical structure of all nanocomposite cryogels. The FT-IR spectra of all samples were recorded on a Bruker Vertex FT-IR spectrometer (Bruker, Ettlingen, Germany) in the 4000–400 cm^−1^ range, with a resolution of 2 cm^−1^, by the KBr pellet technique, the amount of sample being about 5 mg. Prior to the analysis, the CSGA–zeolite nanocomposite cryogels were first ground under liquid nitrogen and dried under a vacuum in the presence of P_2_O_5_.

#### 4.2.4. SEM, EDX, and Pore-Size Analysis

The cross-sectional microstructure of the CSGA–zeolite nanocomposite cryogels was analyzed with a Verios G4 UC Scanning electron microscope (Thermo Scientific, Brno, Czech Republic). For SEM investigations the samples were coated with a 6 nm platinum layer using a Leica EM ACE200 Sputter coater prior examination in order to provide electrical conductivity and prevent charge buildup, which can occur during exposure to the electron beam. Before SEM analysis, the dried samples were immersed in liquid nitrogen and were immediately cut with a sharp blade to obtain cross-sections through the cryogels. To determine the elemental composition of CS-based nanocomposite cryogels, a SEM, coupled with Energy Dispersive X-ray (EDX) spectroscopy analyzer (Octane Elect Super SDD detector, AMETEK EDAX, Mahwah, NJ, USA), was used. The SEM analysis was performed in High Vacuum mode, utilizing a secondary electron detector (Everhart-Thornley detector, ETD, Thermo Fisher Scientific, Brno, Czech Republic) at 10 kV acceleration voltages. The average pore sizes of all nanocomposite cryogels were obtained by Image J 1.48 v analyzing software, analyzing three different SEM images per sample and measuring at least 30 pores (voids) for each sample.

#### 4.2.5. Swelling Ratio, SR

The swelling kinetics of CSGA cryogel beads were evaluated by immersing about 0.01 g of dried samples in 15 mL aqueous solution with pH 1.2 for 180 min, at RT. The swollen samples were periodically removed from water and weighed, after wiping the excess solvent using filter paper. The *SR* (g g^−1^) was calculated using Equation (8):

(8) $$SR=\frac{W_{t}}{W_{d}}$$

where *W_t_* is the weight of swollen nanocomposite cryogels at time *t*, and *W_d_* is the weight of the freeze-dried cryogels. The measurements were carried out in triplicate, and average data were used for calculation of the *SR*.

#### 4.2.6. Sorption Experiments

**Scheme 1:**
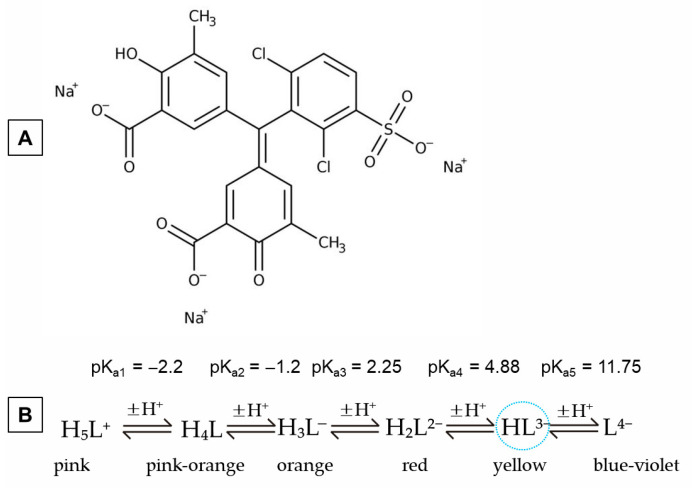
(**A**) Chemical structure of CAS; (**B**) its dissociation species with the corresponding pK_a_ values and associated colors [29,69]. The highlighted species (the blue circle), HL^3−^, corresponds to the depicted chemical structure.

The sorption of CAS dye onto CS–zeolite nanocomposite cryogels was carried out in a batch equilibrium procedure. Isotherm experiments for dye removal were performed at 25 °C by immersing 0.02 g of dried samples in 10 mL of dye solution, the initial solution pH being 5.3. The sorption pH was set to 5.3, as previous studies have demonstrated that sorbents of this type efficiently adsorb various pollutants under these conditions [46,47,50]. Moreover, at pH 5.3, CAS is fully ionized according to its ionization scheme, which indicates that the dye can exist in six different structural forms depending on solution pH, as illustrated in Scheme 1 [29,69].

The initial concentrations of the dye ranged from 50 to 1200 mg L^−1^ for CAS. The flasks containing the dye solutions and nanocomposite cryogels were placed on a magnetic plate with multiple positions and stirred at approximately 180 rpm for 24 h. After 24 h, cryogels were filtered off and the residual concentration of the dye remained in the filtrate was measured by the UV–vis spectroscopy, at 453 nm, using a UV–vis SPECORD200, Carl Zeiss, Jena, Germany. The amount of dye sorbed at equilibrium on CS-based cryogels, in mg/g, was obtained using Equation (9):

(9) $$q_{e}=\frac{(C_{0}-C_{e})\;\times \;V}{W}$$

where *C*_0_ and *C_e_* are the dye concentrations (mg L^−1^) before and after the addition of nanocomposite cryogels, respectively, *V* corresponds to the volume of aqueous solution (L), and *W* is the dosage of sorbent (g). For each sorption experiments, the average of three replicates was reported.

The nonlinear isotherm model fittings were performed using OriginPro8.5 software.

#### 4.2.7. Sorption of CAS in the Presence of Competing Ions

The sorption performance of CAS dye by CSGA10 nanocomposite cryogels with varying zeolite contents was further evaluated in the presence of 0.1 M coexisting cations (Na^+^ and Ca^2+^, introduced as NaCl and CaCl_2_, respectively). To assess the effect of these competing ions on sorption capacity, 10 mL of an aqueous CAS dye solution (50 mg L^−1^, prepared in 0.1 M NaCl or 0.1 M CaCl_2_) was added to a flask containing ~0.02 g of dried nanocomposite cryogels. The mixtures were maintained at 25 °C under stirring (180 rpm) for 24 h. After sorption, the residual CAS concentration was determined by UV–vis spectroscopy at 432 nm (NaCl medium) and 433 nm (CaCl_2_ medium). The amount of CAS dye adsorbed onto the CSGA nanocomposite cryogels was then calculated using Equation (9).

#### 4.2.8. Desorption of CAS Dye

The desorption of CAS from the CSGA–zeolite nanocomposite cryogels after a single adsorption cycle was carried out using a two-step chemical treatment to ensure efficient removal of the dye and prepare the materials for subsequent reuse.

The cryogels were first treated with 0.1 M HCl (total volume 20 mL) for 2 h. Following this, the samples were immersed in 50% methanol (10 mL) for approximately 20 h. After solvent treatment, the cryogels were washed three times with distilled water to remove residual acid and methanol, bringing the material close to neutral pH. In the second step, the cryogels were treated five times with 0.1 M NaOH (total volume 50 mL, each cycle 2 h 30 min). After the alkaline treatment, the samples were thoroughly washed with distilled water until neutral pH was reached. Following these steps, the cryogels were fully regenerated and ready for a new cycle of sorption/desorption.

#### 4.2.9. Reusability Study

The reusability of the CSGA10, CSGA10Z20, and CSGA10Z40 nanocomposite cryogels was evaluated using a batch equilibrium procedure. Sorption–desorption experiments were conducted over three consecutive cycles at 25 °C. For each cycle, approximately 0.02 g of dried nanocomposite beads was immersed in 10 mL of CAS solution (50 mg L^−1^). The vials containing the cryogels and dye solution were placed on a multi-position magnetic stirrer and stirred at 180 rpm for 24 h. After equilibration, the CAS-loaded sorbents were separated by decantation, and the supernatant was analyzed by UV–vis spectrophotometry at 453 nm. The equilibrium adsorption capacity of CAS dye on the nanocomposite cryogels (mg g^−1^) was then calculated according to Equation (9).

## Figures and Tables

**Table 1:** Composition, GFY, and average pore sizes of the CSGA nanocomposite cryogels at different cross-linker ratios and zeolite contents.

Sample Code	GA, wt.%	CPL, wt.%	^a^ GFY, % ± SD	^b^ Diameter of Beads, mm
CSGA5	5	0	86.00 ± 0.61	3.00 ± 0.09
CSGA5Z10	5	10	85.04 ± 0.80	2.90 ± 0.12
CSGA5Z20	5	20	84.74 ± 0.60	3.03 ± 0.03
CSGA5Z40	5	40	85.30 ± 0.87	2.93 ± 0.12
CSGA7.5	7.5	0	82.55 ± 0.49	2.88 ± 0.13
CSGA7.5Z10	7.5	10	86.61 ± 0.43	2.57 ± 0.11
CSGA7.5Z20	7.5	20	88.17 ± 1.42	2.94 ± 0.11
CSGA7.5Z40	7.5	40	83.99 ± 0.25	2.8 ± 0.09
CSGA10	10	0	81.28 ± 0.97	2.69 ± 0.06
CSGA10Z10	10	10	82.91 ± 0.65	2.77 ± 0.09
CSGA10Z20	10	20	81.21 ± 1.41	2.88 ± 0.06
CSGA10Z40	10	40	82.19 ± 0.08	2.68 ± 0.03

^a^ Gel fraction yield (GFY) was calculated with Equation (7); ^b^ The diameters of beads were evaluated with a digital caliper.

**Table 2:** Kinetic model parameters for water absorption by the CSGA–zeolite nanocomposite cryogels.

Sample	PFO Kinetic Model			PSO Kinetic Model		
	*k*_1_ (min^−1^)	*q_e_* (g/g)	R^2^	*k*_2_ (g/g·min)	*q_e_* (g/g)	R^2^
CSGA5	0.569	60.120	0.999	0.059	60.565	0.999
CSGA5Z10	0.757	47.664	0.996	0.101	48.014	0.997
CSGA5Z20	0.669	42.924	0.996	0.074	43.391	0.998
CSGA5Z40	0.734	39.961	0.997	0.104	40.304	0.999
CSGA7.5	0.897	51.747	0.999	0.204	51.920	0.999
CSGA7.5Z10	0.889	47.277	0.999	0.224	47.433	0.999
CSGA7.5Z20	0.545	43.807	0.996	0.055	44.354	0.997
CSGA7.5Z40	0.578	38.340	0.999	0.078	38.719	0.999
CSGA10	0.653	49.567	0.999	0.116	49.790	0.999
CSGA10Z10	0.631	46.308	0.999	0.085	46.661	0.999
CSGA10Z20	0.645	41.790	0.998	0.092	42.132	0.999
CSGA10Z40	0.612	36.601	0.998	0.129	36.799	0.998

**Table 3:** Isotherms fitting parameters for CAS adsorption by the CSGA5 nanocomposite cryogels with different zeolite contents.

Isotherm Model	Parameters	CSGA5	CSGA5Z10	CSGA5Z20	CSGA5Z40
Langmuir	*q_m,exp_*, mg/g	117.948	182.811	213.017	250.811
	*q_m_*, mg/g	109.516	164.438	188.6	249.607
	*K_L_*, L/mg	0.561	0.41	0.29	0.012
	R^2^	0.946	0.936	0.905	0.815
	χ^2^	91.666	275.571	553.38	1431.725
Freundlich	*K_F_*, L/mg	45.192	59.789	55.578	52.954
	1/*n*	0.144	0.161	0.192	0.219
	R^2^	0.964	0.935	0.955	0.964
	χ^2^	60.74	281.204	261.301	274.186
D-R	*q_DR_*, mg/g	107.638	157.241	180.301	199.643
	*β*	0.147	0.13	0.246	0.238
	*E*, kJ/mol	1.844	1.961	1.426	1.449
	R^2^	0.917	0.867	0.839	0.799
	χ^2^	141.431	571.677	942.823	1552.938

**Table 4:** Isotherms fitting parameters for CAS adsorption by the CSGA7.5 nanocomposite cryogels with different zeolite contents.

Isotherm Model	Parameters	CSGA7.5	CSGA7.5Z10	CSGA7.5Z20	CSGA7.5Z40
Langmuir	*q_m,exp_*, mg/g	199.934	220.483	208.851	196.26
	*q_m_*, mg/g	188.933	221.704	204.294	178.926
	*K_L_*, L/mg	0.804	0.052	0.134	1.725
	R^2^	0.85	0.731	0.744	0.901
	χ^2^	957.013	2088.811	1857.328	601.459
Freundlich	*K_F_*, L/mg	103.775	106.695	130.416	103.481
	1/*n*	0.108	0.115	0.073	0.098
	R^2^	0.855	0.732	0.746	0.914
	χ^2^	922.727	2076.453	1844.613	521.128
D-R	*q_DR_*, mg/g	187.238	200.643	193.191	178.214
	*β*	0.209	2.938	0.046	0.047
	*E*, kJ/mol	1.547	0.413	3.297	3.262
	R^2^	0.847	0.236	0.234	0.899
	χ^2^	973.926	348.922	159.877	608.907

**Table 5:** Isotherms fitting parameters for CAS adsorption by the CSGA10 nanocomposite cryogels with different zeolite contents.

Isotherm Model	Parameters	CSGA10	CSGA10Z10	CSGA10Z20	CSGA10Z40
Langmuir	*q_m,exp_*, mg/g	185.074	182.053	180.889	164.844
	*q_m_*, mg/g	169.276	174.773	165.536	146.274
	*K_L_*, L/mg	1.27	1.041	1.36	0.276
	R^2^	0.894	0.9	0.881	0.833
	χ^2^	576.069	564.914	603.454	624.377
Freundlich	*K_F_*, L/mg	91.919	99.374	95.409	74.519
	1/*n*	0.107	0.099	0.097	0.114
	**R^2^**	**0.913**	**0.902**	**0.898**	**0.854**
	χ^2^	472.95	551.749	516.242	545.899
D-R	*q_DR_*, mg/g	168.633	173.841	164.901	144.16
	*β*	0.087	0.133	0.092	2.829
	*E*, kJ/mol	2.397	1.939	2.331	0.420
	R^2^	0.893	0.899	0.88	0.83
	χ^2^	582.045	571.226	609.082	639.033

## Data Availability

Data are contained within the article and Supplementary Materials.

## Supplementary Materials

The following supporting information can be downloaded at: https://www.mdpi.com/article/10.3390/gels11090729/s1, Figure S1. SEM micrograph of CSGA5Z40 nanocomposite cryogels at a magnification of 1000×. Figure S2. SEM micrographs of CSGA7.5Z20 nanocomposite cryogels at magnifications of 500× (A) and 1000× (B), respectively. Figure S3. EDX spectra of CSGA5 nanocomposite cryogels with different zeolite contents. Figure S4. EDX spectra of CSGA7.5 nanocomposite cryogels with different zeolite contents. Figure S5. EDX spectra of CSGA10 nanocomposite cryogels with different zeolite contents. Figure S6. FTIR spectra of CSGA5 nanocomposite cryogels with different zeolite contents. Figure S7. FTIR spectra of CSGA7.5 nanocomposite cryogels with different zeolite contents. Figure S8. FTIR spectra of CS, zeolite (clinoptilolite), and CAS dye. Figure S9. Langmuir, Freundlich, and Dubinin–Radushkevich (DR) isotherm profiles for the adsorption of CAS by CSGA5 nanocomposite cryogels with different zeolite contents. Figure S10. Langmuir, Freundlich, and Dubinin–Radushkevich (DR) isotherm profiles for the adsorption of CAS by CSGA7.5 nanocomposite cryogels with different zeolite contents. Figure S11. Langmuir, Freundlich, and Dubinin–Radushkevich (DR) isotherm profiles for the adsorption of CAS by CSGA5 nanocomposite cryogels with different zeolite contents. Figure S12. EDX spectra of CSGA5 nanocomposite cryogels with different zeolite contents after sorption of CAS. Figure S13. EDX spectra of CSGA7.5 nanocomposite cryogels with different zeolite contents after sorption of CAS. Figure S14. EDX spectra of CSGA10 nanocomposite cryogels with different zeolite contents after sorption of CAS. Figure S15. FTIR spectra of CAS-loaded CSGA5 nanocomposite cryogels with different zeolite contents. Figure S16. FTIR spectra of CAS-loaded CSGA7.5 nanocomposite cryogels with different zeolite contents. Figure S17. FTIR of CSGA10, CSGA10Z20, and CSGA10Z40 sorbents after regeneration and 3rd cycle of reuse. Table S1. Ca^2+^ and K^+^ atomic percent before and after CAS sorption.
