# Supplementary material for: Nanocomposite Cryogels Based on Chitosan for Efficient Removal of a Triphenylmethane Dye from Aqueous Systems
Source: Gels. 2025 Sep 11;11(9):729. doi: 10.3390/gels11090729 (PMC12470090; doi:10.3390/gels11090729)
Supplement: Supplementary file 1 [file gels-11-00729-s001.zip › gels-3833560-supplementary.pdf]

Supporting material

Article

Nanocomposite Cryogels Based on Chitosan for Efficient Removal of a Triphenylmethane Dye from Aqueous Systems

Maria Marinela Lazar, Claudiu Augustin Ghiorghita, Daniela Rusu, MariaValentina Dinu*

"Petru Poni" Institute of Macromolecular Chemistry, Grigore Ghica Voda Alley 41A, Iasi 700487, Romania;

* Correspondence: vdinu@icmpp.ro

Contents

Figure S1. SEM micrograph of CSGA5Z40 nanocomposite cryogels at magnification of 1000x.

Figure S2. SEM micrographs of CSGA7.5Z20 nanocomposite cryogels at magnification of 500x (A) and respectively, 1000x (B).

Figure S3. EDX spectra of CSGA5 nanocomposite cryogels with different zeolite content.

Figure S4. EDX spectra of CSGA7.5 nanocomposite cryogels with different zeolite content.

Figure S5. EDX spectra of CSGA10 nanocomposite cryogels with different zeolite content.

Figure S6. FTIR spectra of CSGA5 nanocomposite cryogels with different zeolite content.

Figure S7. FTIR spectra of CSGA7.5 nanocomposite cryogels with different zeolite content.

Figure S8. FTIR spectra of CS, zeolite (clinoptilolite), and CAS dye.

Figure S9. Langmuir, Freundlich, and Dubinin-Radushkevich (DR) isotherm profiles for the adsorption of CAS by CSGA5 nanocomposite cryogels with different zeolite content.

Figure S10. Langmuir, Freundlich, and Dubinin-Radushkevich (DR) isotherm profiles for the adsorption of CAS by CSGA7.5 nanocomposite cryogels with different zeolite content.

Figure S11. Langmuir, Freundlich, and Dubinin-Radushkevich (DR) isotherm profiles for the adsorption of CAS by CSGA5 nanocomposite cryogels with different zeolite content.

Figure S12. EDX spectra of CSGA5 nanocomposite cryogels with different zeolite content after sorption of CAS.

Figure S13. EDX spectra of CSGA7.5 nanocomposite cryogels with different zeolite content after sorption of CAS.

Figure S14. EDX spectra of CSGA10 nanocomposite cryogels with different zeolite content after sorption of CAS.

Figure S15. FTIR spectra of CAS-loaded CSGA5 nanocomposite cryogels with different zeolite content.

Figure S16. FTIR spectra of CAS-loaded CSGA7.5 nanocomposite cryogels with different zeolite content.

Figure S17. FTIR of CSGA10, CSGA10Z20, and CSGA10Z40 sorbents after regeneration and 3rd cycle of reuse.

Table S1. Ca^{2+} and K^{+} atomic percent before and after CAS sorption.

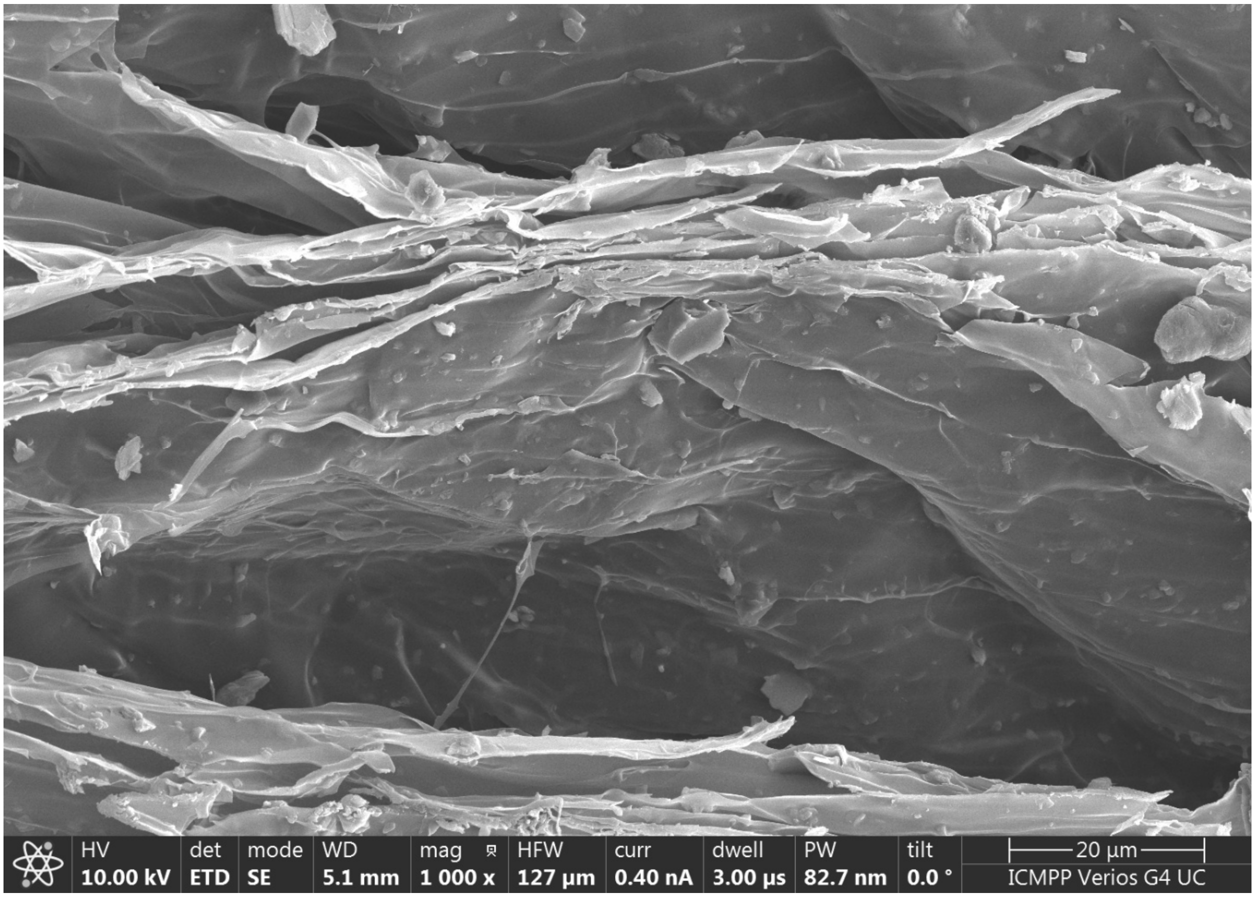

Figure S1. SEM micrographs of CSGA5Z40 nanocomposite cryogels at magnification of 1000x.

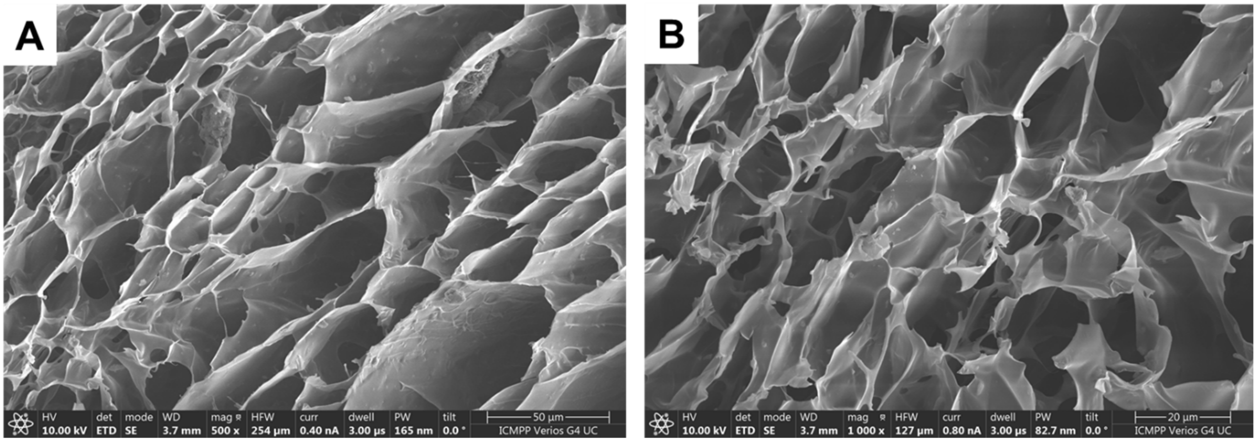

Figure S2. SEM micrograph of CSGA7.5Z20 nanocomposite cryogels at magnification of 500x (A) and respectively, 1000x (B).

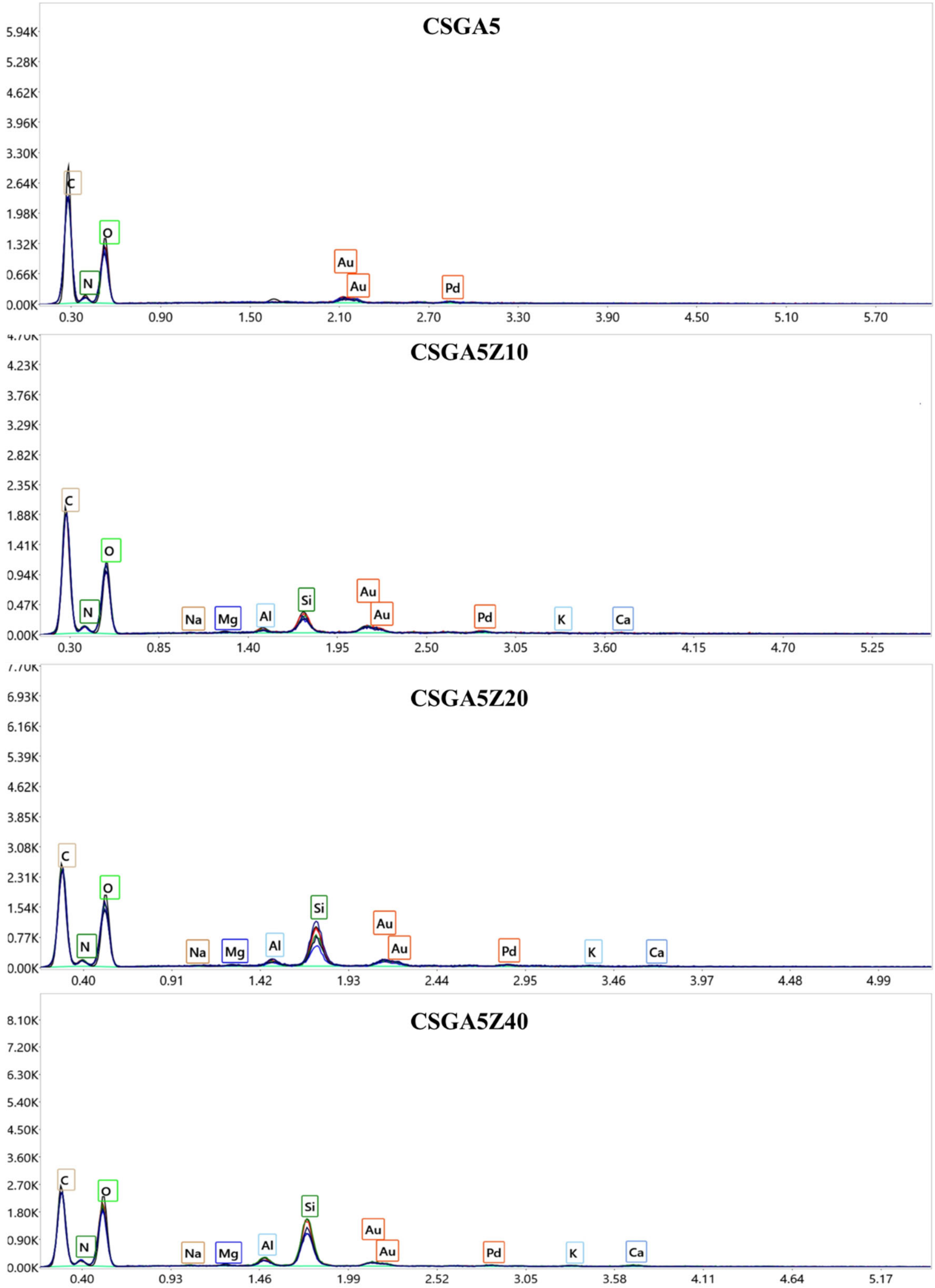

Figure S3. EDX spectra of CSGA5 nanocomposite cryogels with different zeolite content.

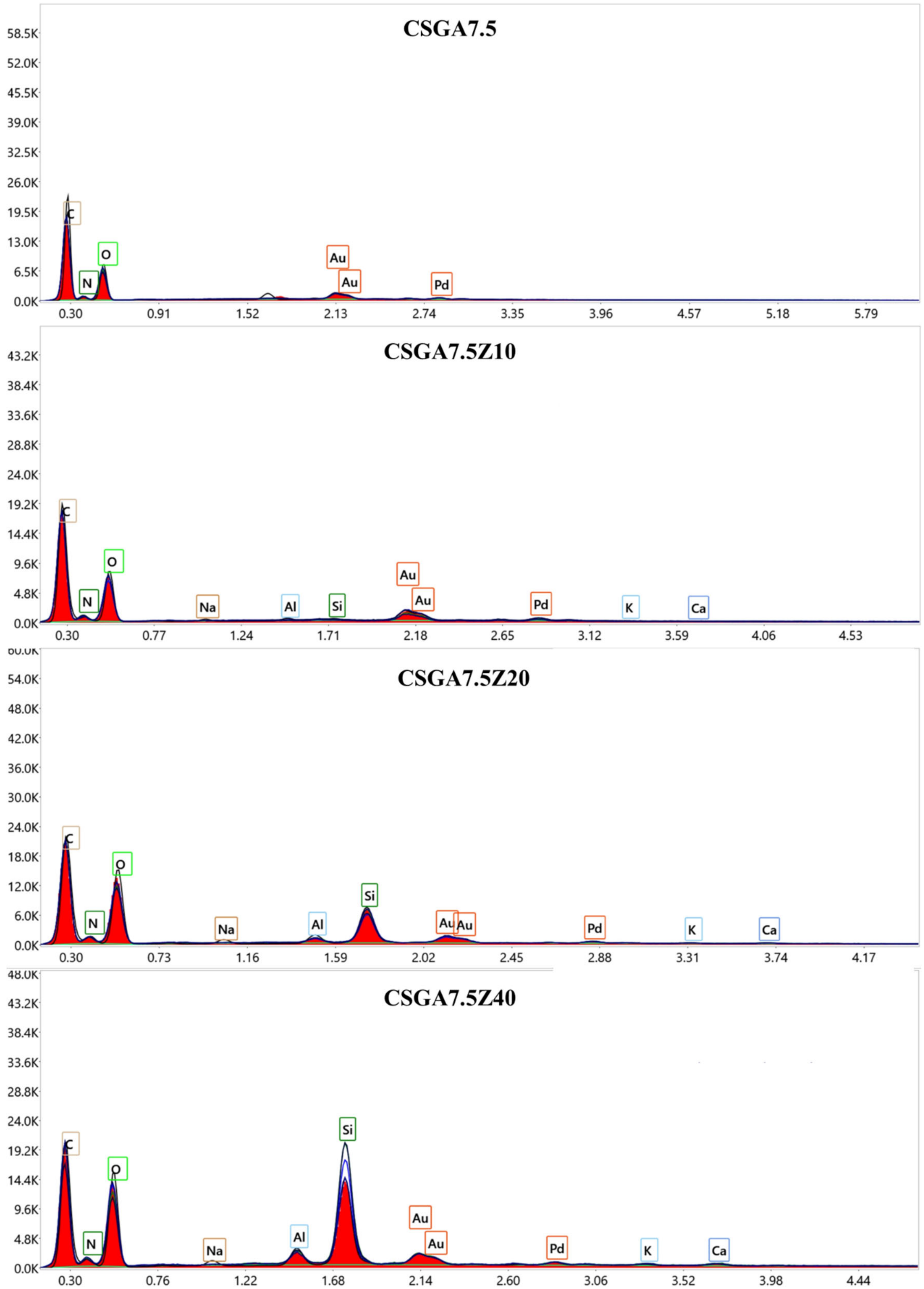

Figure S4. EDX spectra of CSGA7.5 nanocomposite cryogels with different zeolite content.

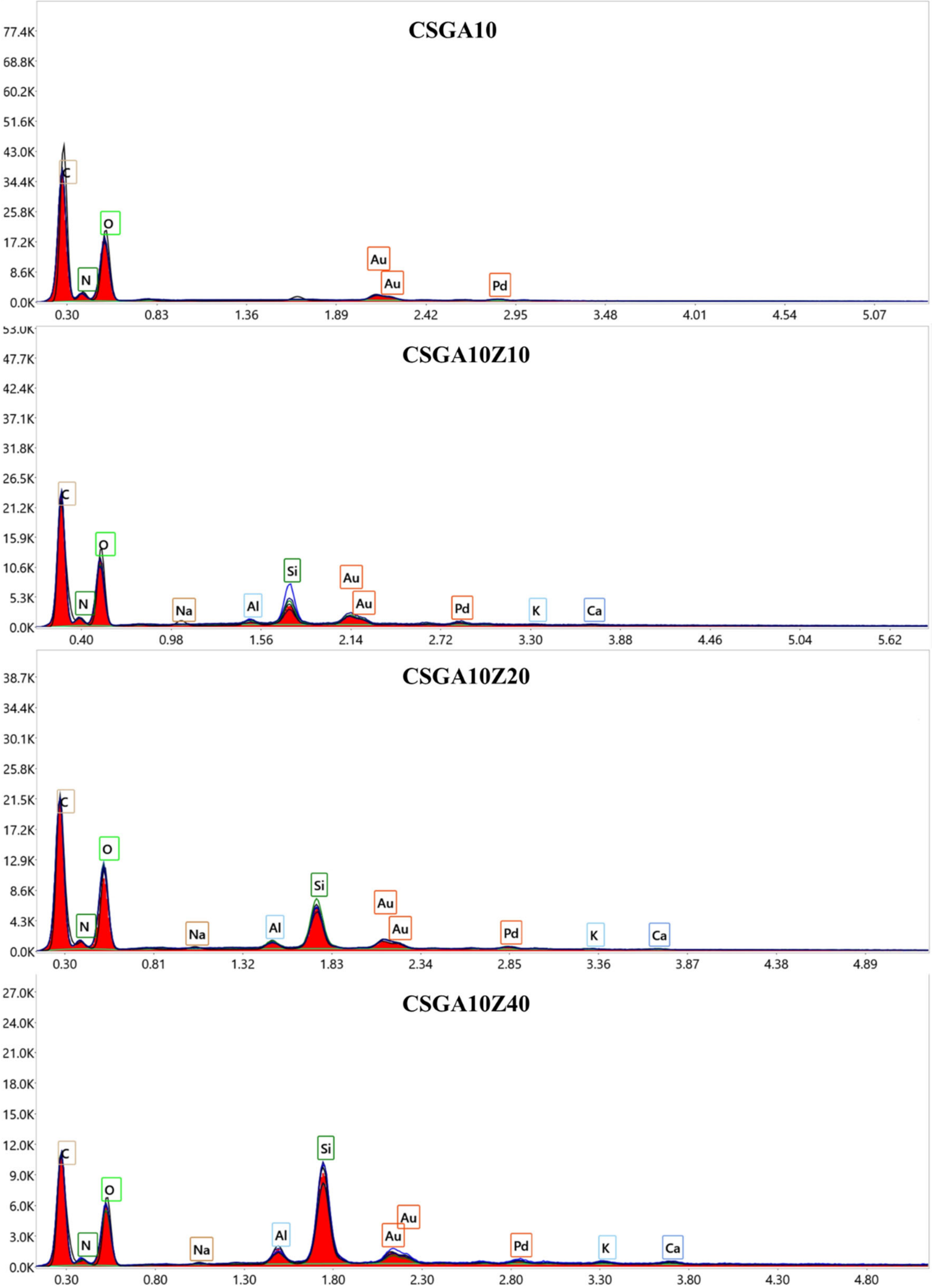

Figure S5. EDX spectra of CSGA nanocomposite cryogels with different zeolite content.

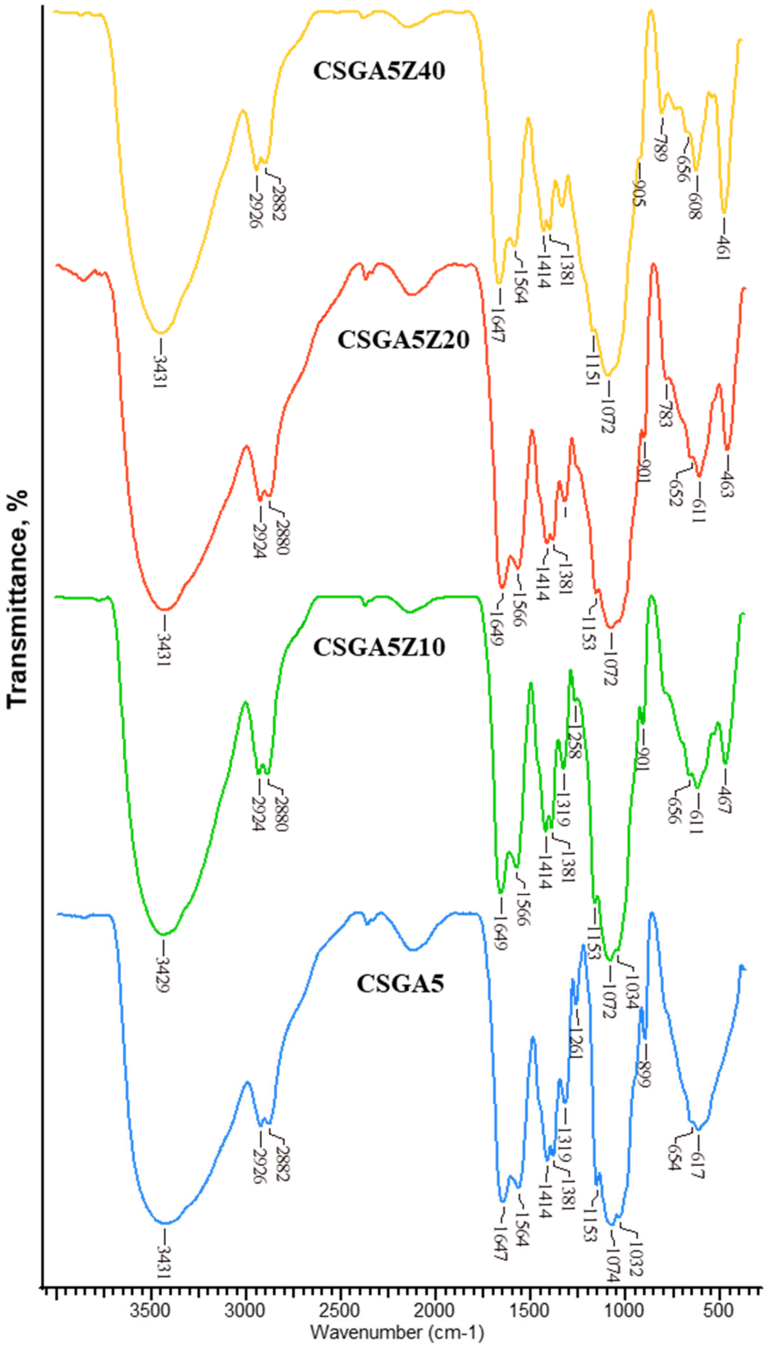

Figure S6. FTIR spectra of CSGA5 nanocomposite cryogels with different zeolite content.

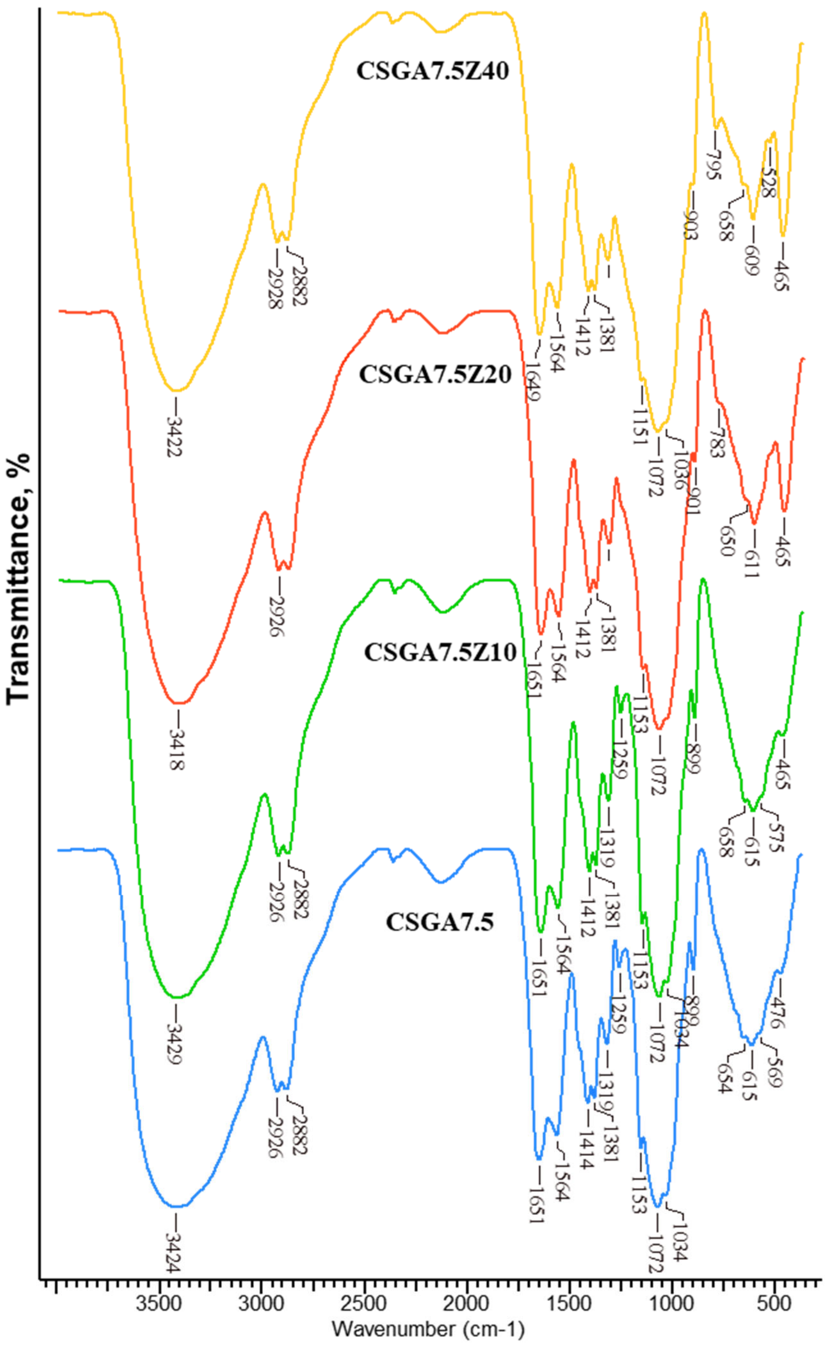

Figure S7. FTIR spectra of CSGA7.5 nanocomposite cryogels with different zeolite content.

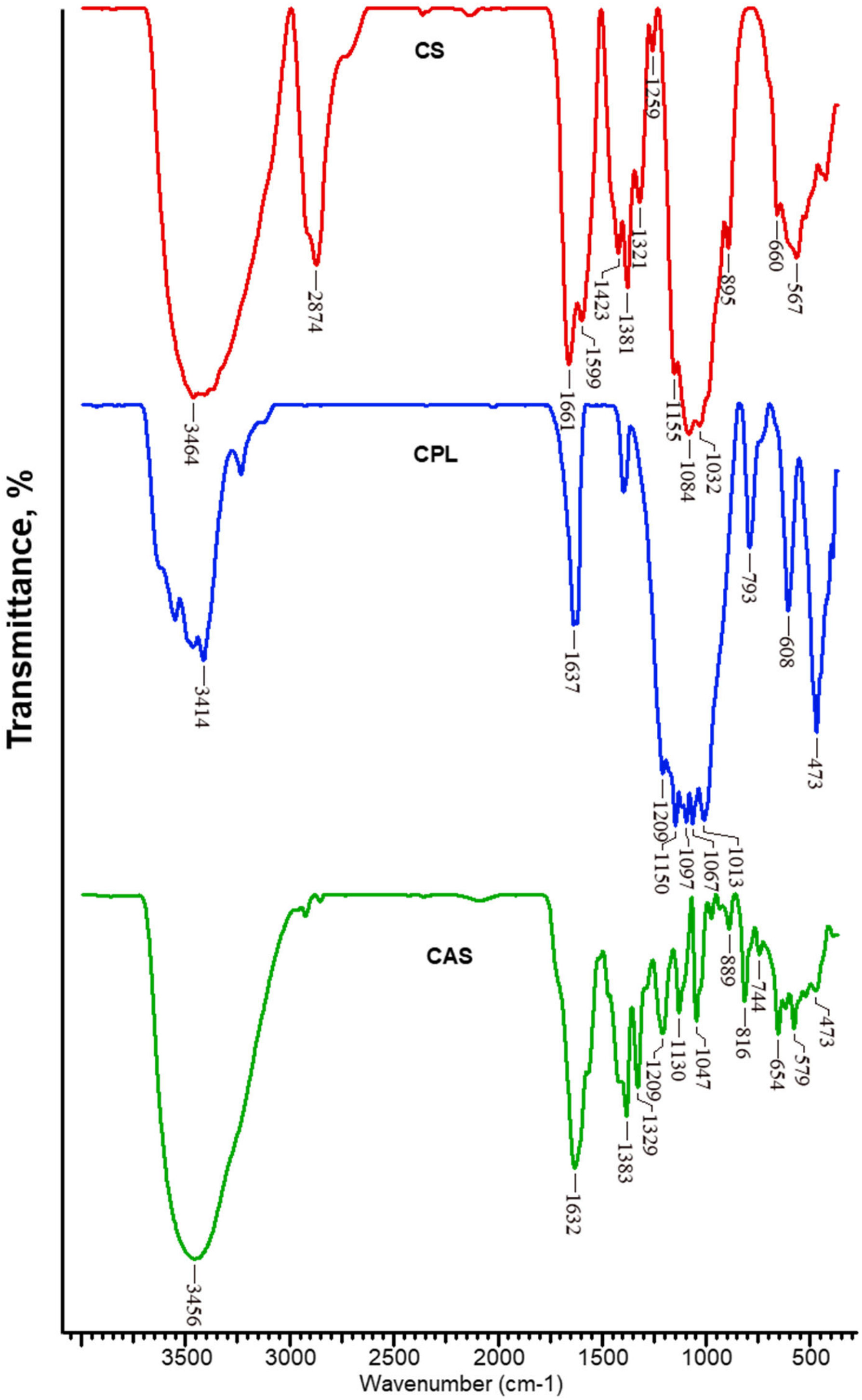

Figure S8. FTIR spectra of CS, zeolite (clinoptilolite), and CAS dye.

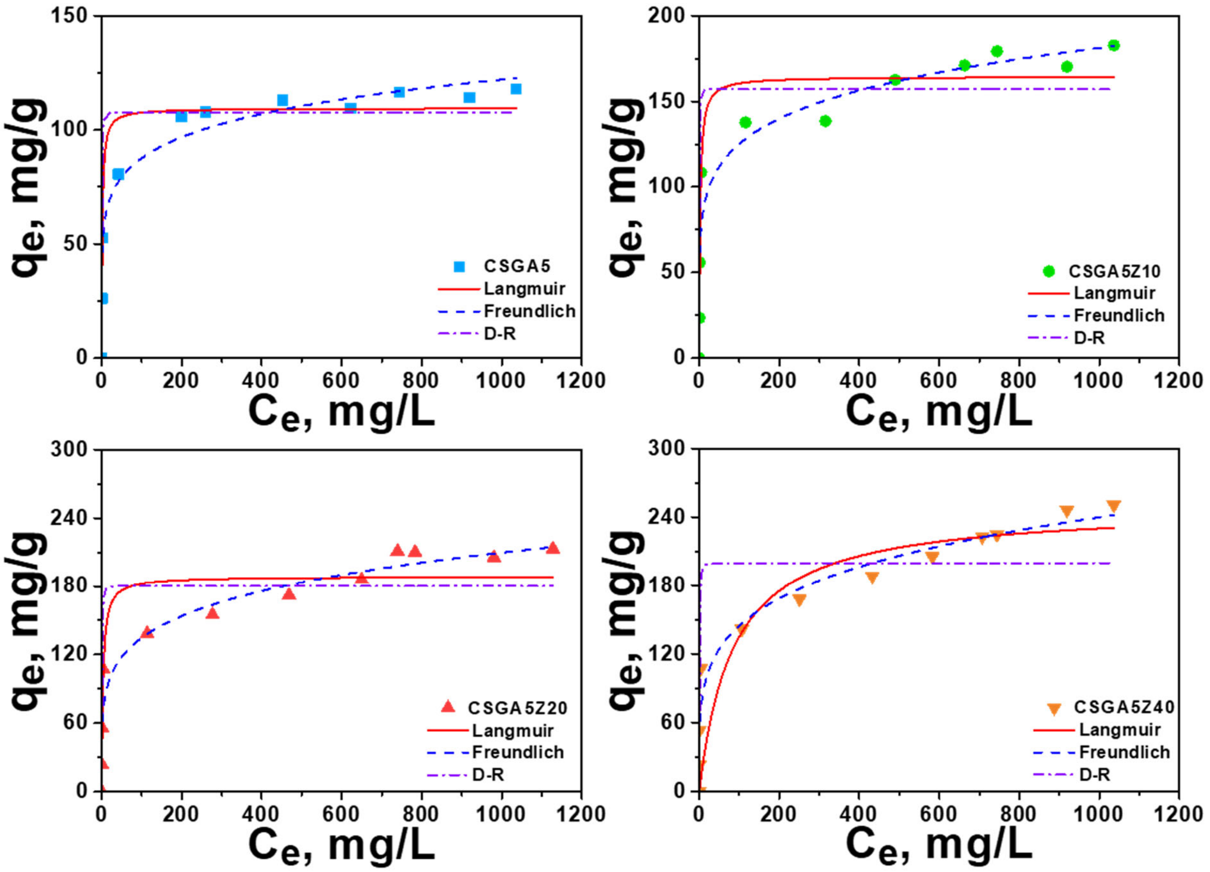

Figure S9. Langmuir, Freundlich, and Dubinin-Radushkevich (DR) isotherm profiles for the adsorption of CAS by CSGA5 nanocomposite cryogels with different zeolite content.

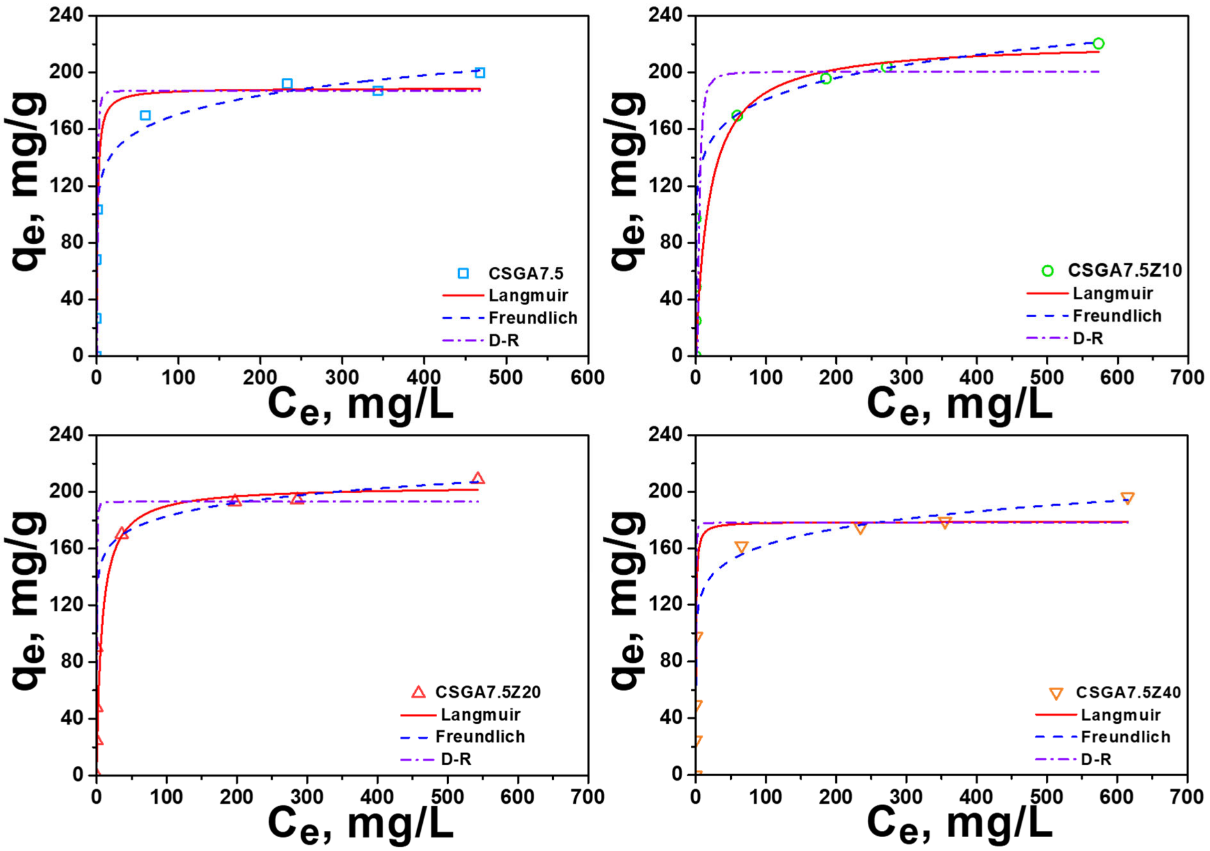

Figure S10. Langmuir, Freundlich, and Dubinin-Radushkevich (DR) isotherm profiles for the adsorption of CAS by CSGA7.5 nanocomposite cryogels with different zeolite content.

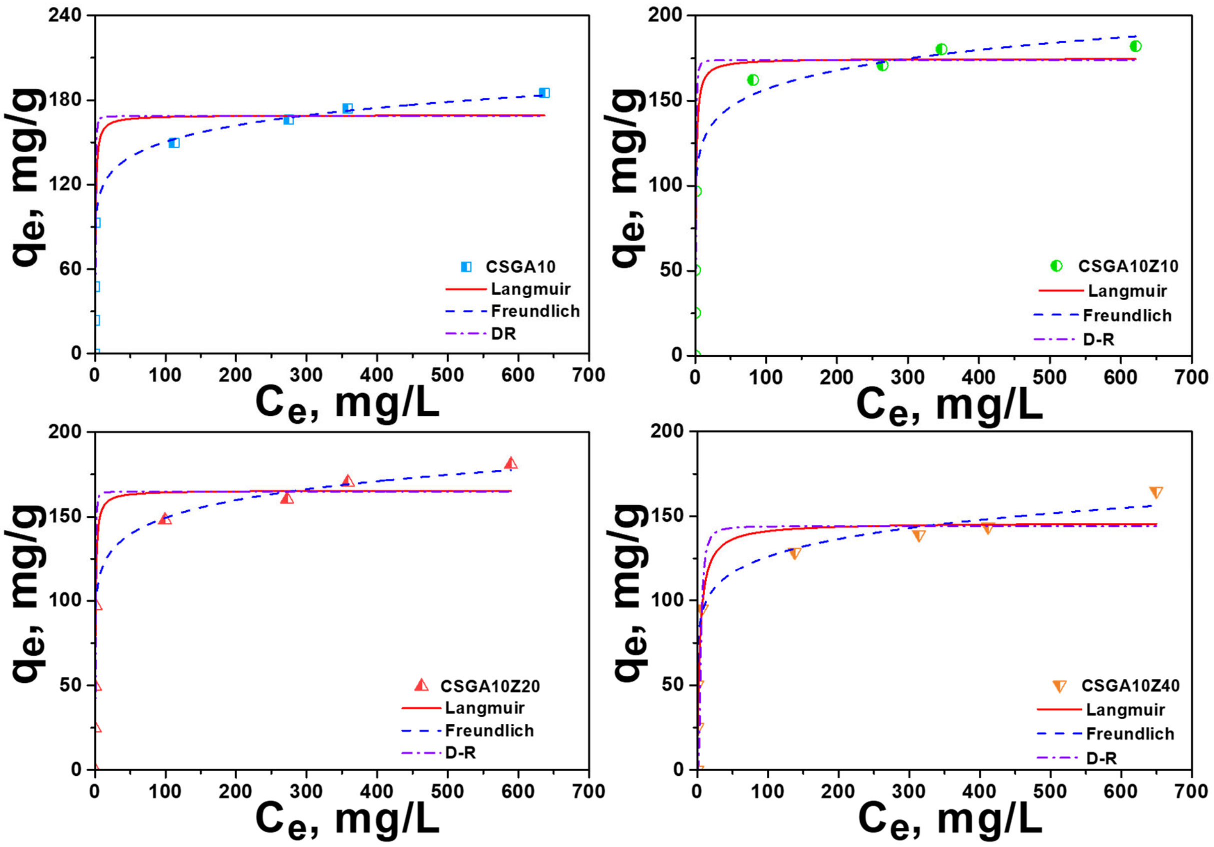

Figure S11. Langmuir, Freundlich, and Dubinin-Radushkevich (DR) isotherm profiles for the adsorption of CAS by CSGA10 nanocomposite cryogels with different zeolite content.

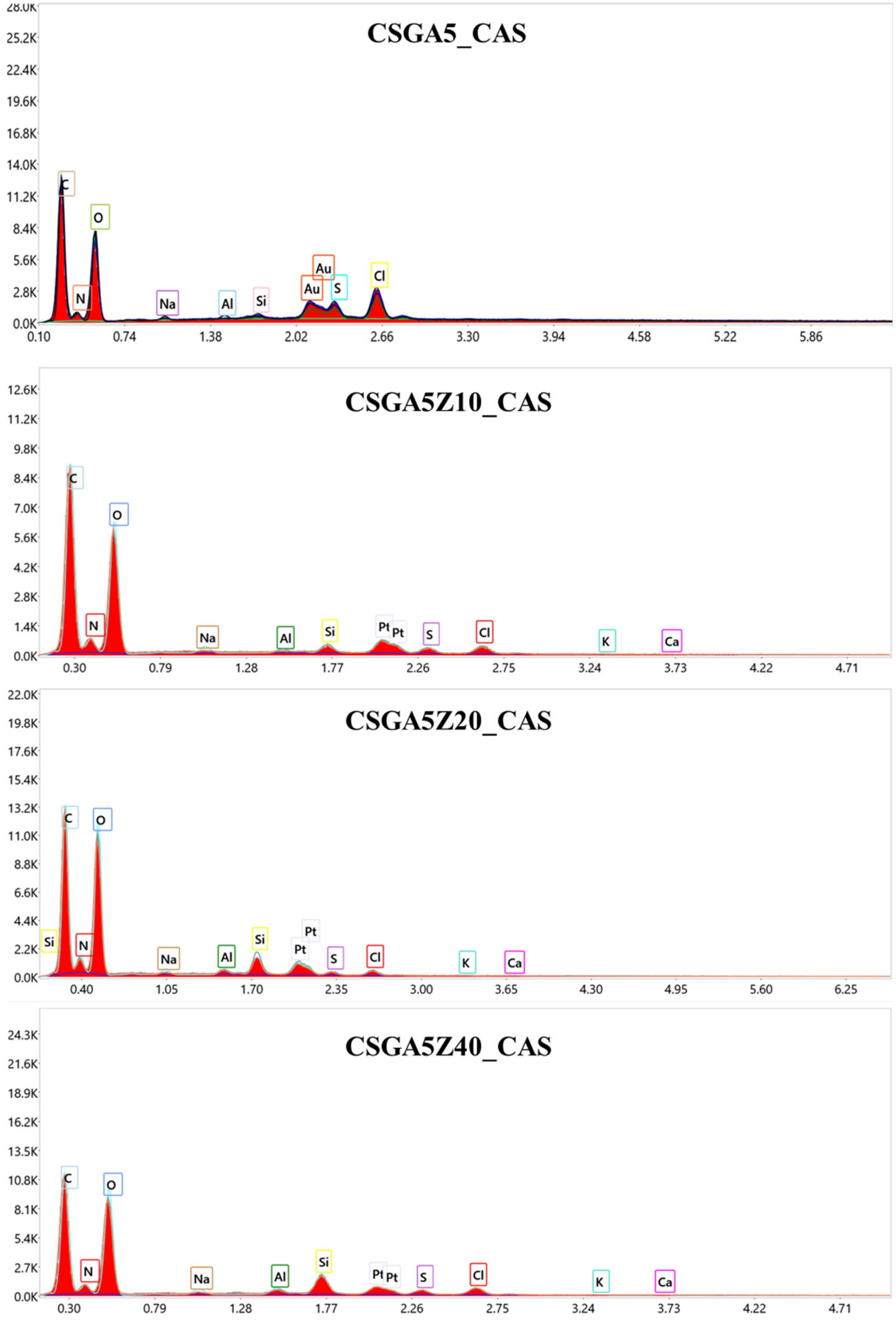

Figure S12. EDX spectra of CSGA5 nanocomposite cryogels with different zeolite content after sorption of CAS.

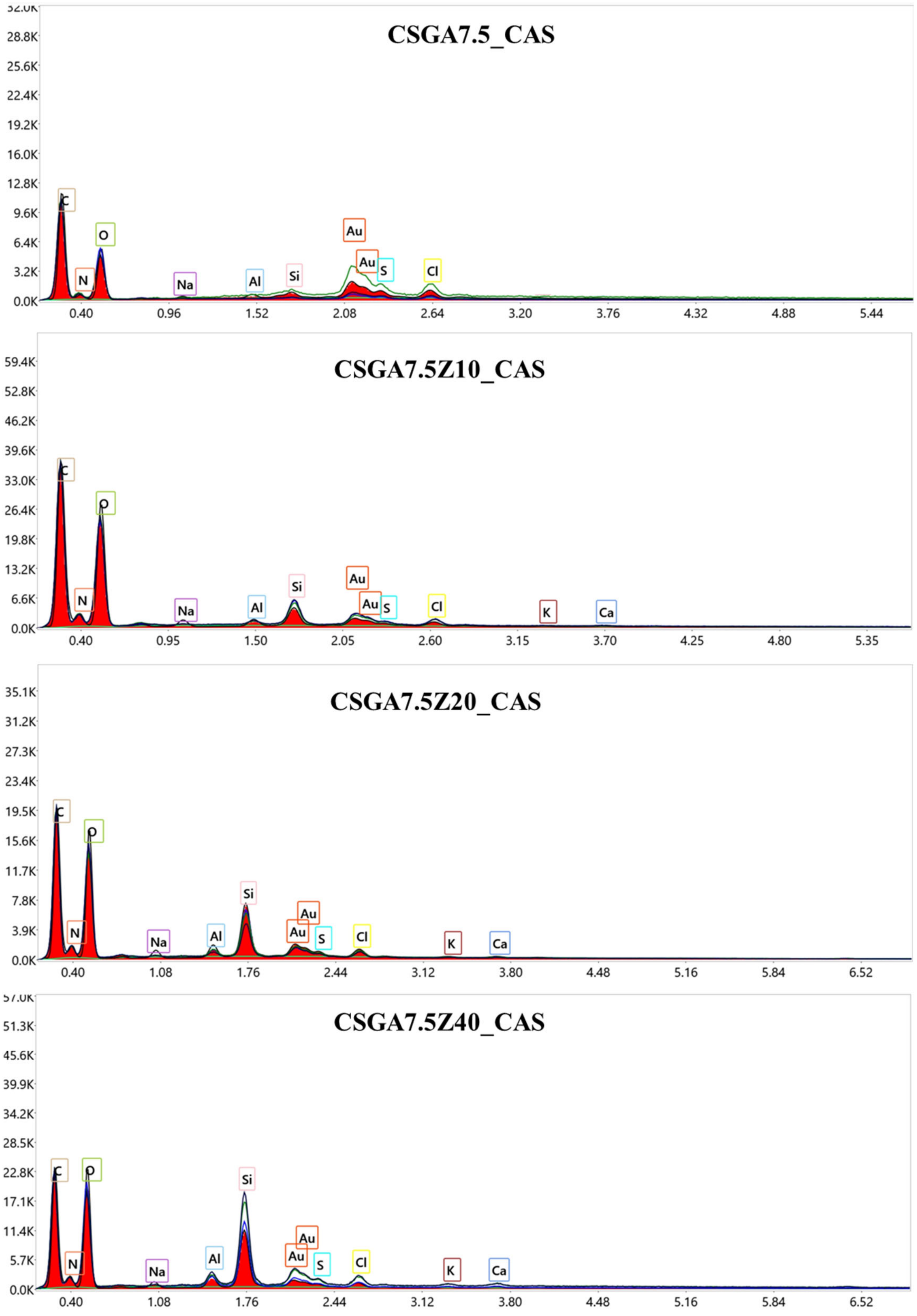

Figure S13. EDX spectra of CSGA7.5 nanocomposite cryogels with different zeolite content after sorption of CAS.

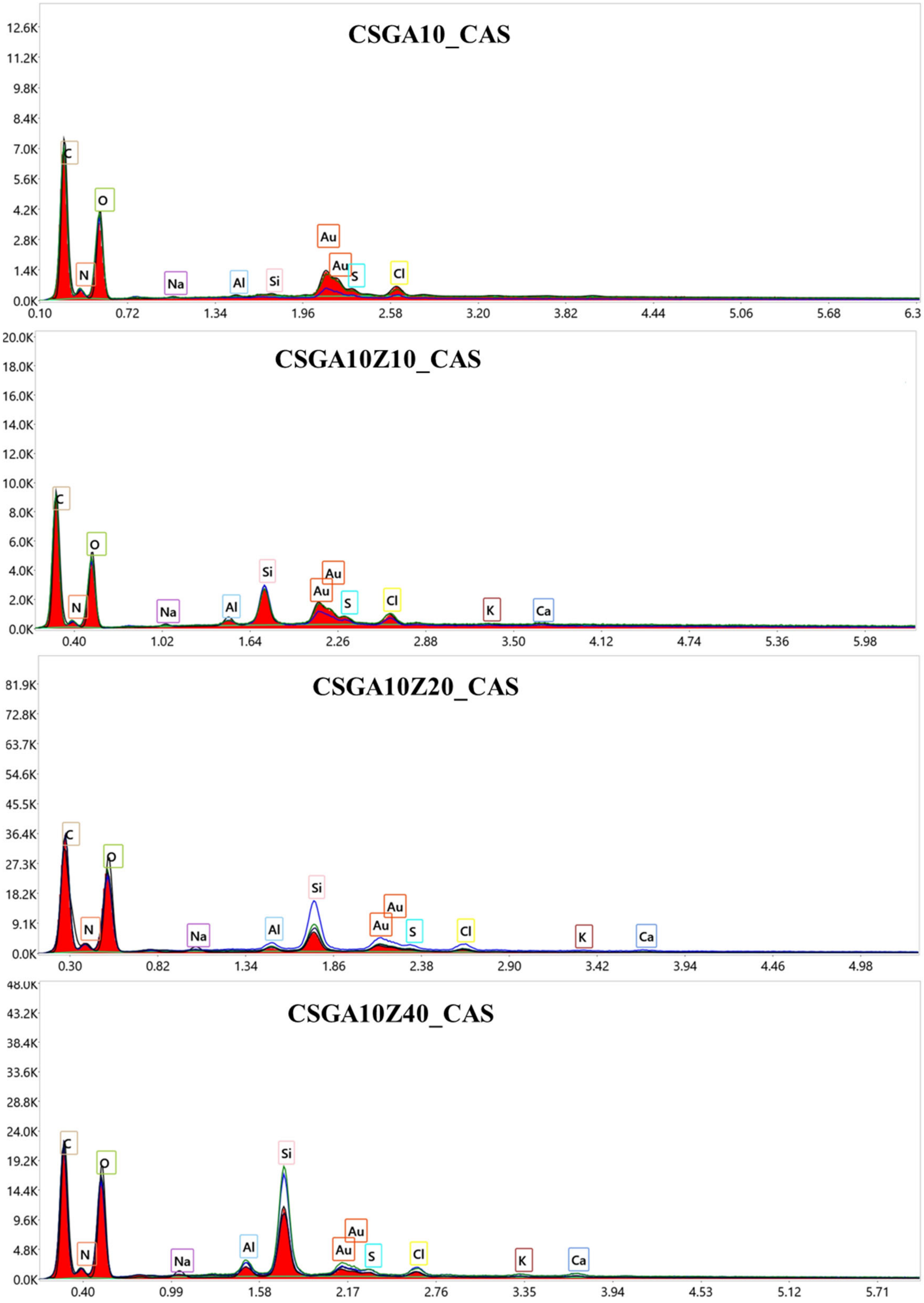

Figure S14. EDX spectra of CSGA10 nanocomposite cryogels with different zeolite content after sorption of CAS.

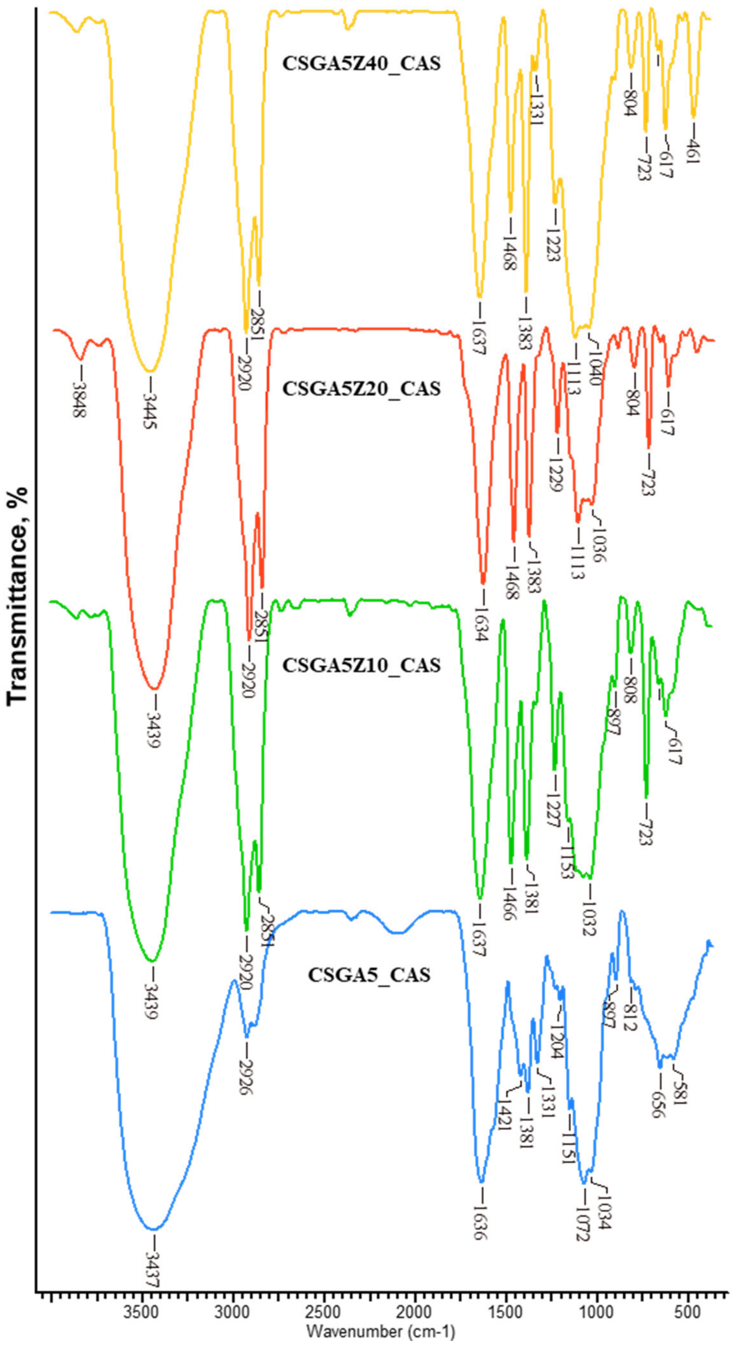

Figure S15. FTIR spectra of CAS-loaded CSGA5 nanocomposite cryogels with different zeolite content.

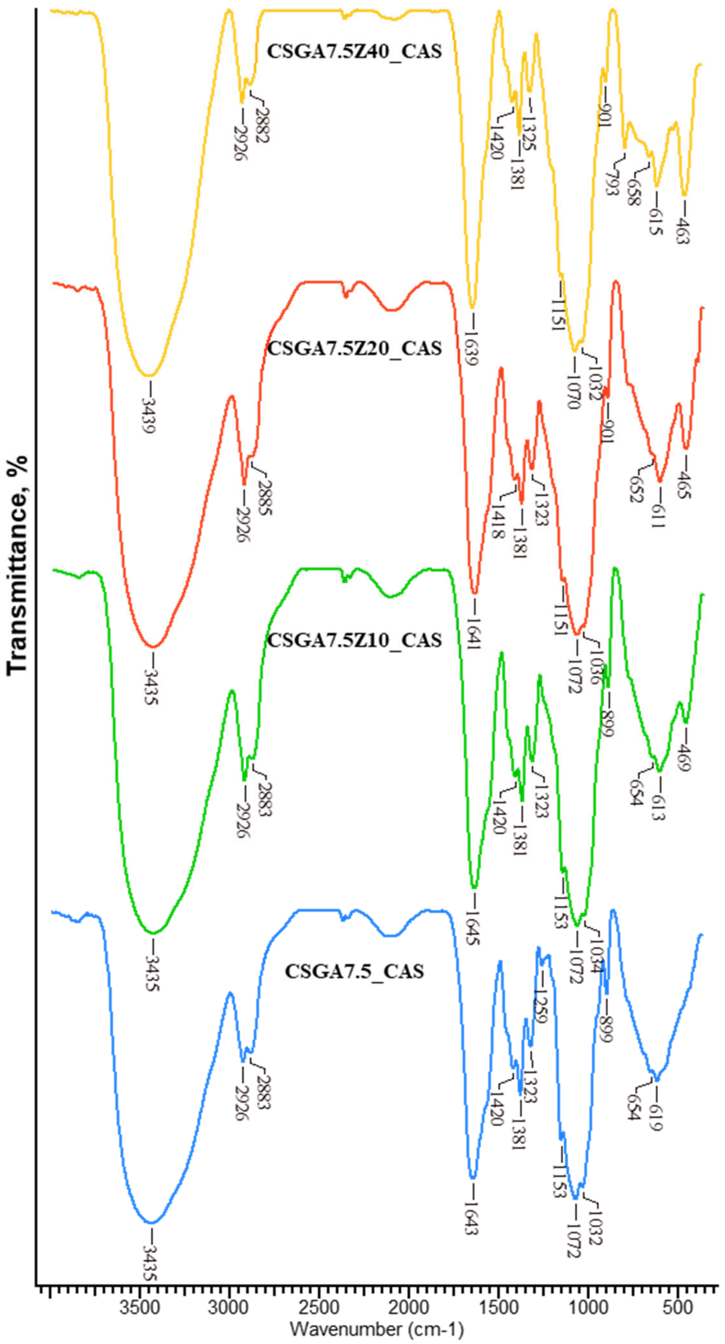

Figure S16. FTIR spectra of CAS-loaded CSGA7.5 nanocomposite cryogels with different zeolite content.

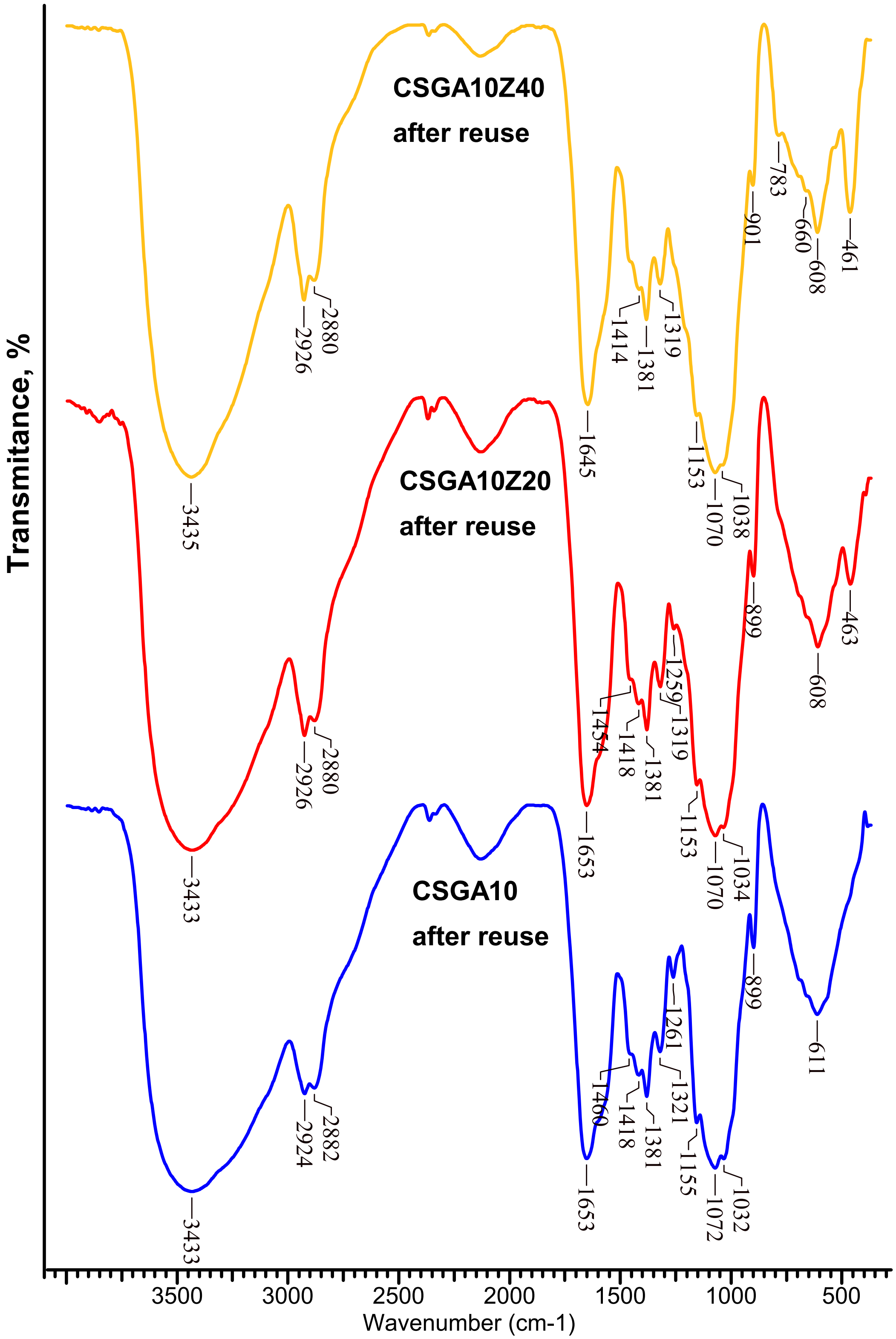

Figure S17. FTIR of CSGA10, CSGA10Z20, and CSGA10Z40 sorbents after regeneration and 3rd cycle of reuse.

Table S1. Ca^{2+} and K^{+} atomic percent before and after CAS sorption.

Sample Code	Ca^{2+}, %		K^{+}, %	
	**Before CAS Sorption**	**After CAS Sorption**	**Before CAS Sorption**	**After CAS Sorption**
**CSGA5**	0	0	0	0
**CSGA5Z10**	0.09 ± 0.008	0.10 ± 0.01	0.06 ± 0.004	0.10 ± 0.01
**CSGA5Z20**	0.13 ± 0.01	0.10 ± 0.02	0.12 ± 0.009	0.10 ± 0.01
**CSGA5Z40**	0.14 ± 0.01	0.13 ± 0.02	0.12 ± 0.02	0.12 ± 0.03
**CSGA7.5**	0	0	0	0
**CSGA7.5Z10**	0.03 ± 0.01	0.03 ± 0.02	0.01 ± 0.004	0.02 ± 0.001
**CSGA7.5Z20**	0.08 ± 0.004	0.04 ± 0.01	0.06 ± 0.02	0.04 ± 0.004
**CSGA7.5Z40**	0.16 ± 0.02	0.16 ± 0.06	0.10 ± 0.02	0.11 ± 0.04
**CSGA10**	0	0	0	0
**CSGA10Z10**	0.05 ± 0.02	0.16 ± 0.03	0.03 ± 0.02	0.09 ± 0.03
**CSGA10Z20**	0.06 ± 0.01	0.05 ± 0.01	0.03 ± 0.01	0.04 ± 0.02
**CSGA10Z40**	0.26 ± 0.03	0.14 ± 0.04	0.14 ± 0.02	0.08 ± 0.02
